# A Deviation from the Bipolar-Tetrapolar Mating Paradigm in an Early Diverged Basidiomycete

**DOI:** 10.1371/journal.pgen.1001052

**Published:** 2010-08-05

**Authors:** Marco A. Coelho, José Paulo Sampaio, Paula Gonçalves

**Affiliations:** Centro de Recursos Microbiológicos (CREM), Departamento de Ciências da Vida, Faculdade de Ciências e Tecnologia, Universidade Nova de Lisboa, Caparica, Portugal; University of Wisconsin-Madison, United States of America

## Abstract

In fungi, sexual identity is determined by specialized genomic regions called *MAT* loci which are the equivalent to sex chromosomes in some animals and plants. Usually, only two sexes or mating types exist, which are determined by two alternate sets of genes (or alleles) at the *MAT* locus (bipolar system). However, in the phylum Basidiomycota, a unique tetrapolar system emerged in which four different mating types are generated per meiosis. This occurs because two functionally distinct molecular recognition systems, each encoded by one *MAT* region, constrain the selection of sexual partners. Heterozygosity at both *MAT* regions is a pre-requisite for mating in both bipolar and tetrapolar basidiomycetes. Tetrapolar mating behaviour results from the absence of genetic linkage between the two regions bringing forth up to thousands of mating types. The subphylum Pucciniomycotina, an early diverged lineage of basidiomycetes encompassing important plant pathogens such as the rusts and saprobes like *Rhodosporidium* and *Sporidiobolus*, has been so far poorly explored concerning the content and organization of *MAT* loci. Here we show that the red yeast *Sporidiobolus salmonicolor* has a mating system unlike any previously described because occasional disruptions of the genetic cohesion of the bipolar *MAT* locus originate new mating types. We confirmed that mating is normally bipolar and that heterozygosity at both *MAT* regions is required for mating. However, a laboratory cross showed that meiotic recombination may occur within the bipolar *MAT* locus, explaining tetrapolar features like increased allele number and evolution rates of some *MAT* genes. This pseudo-bipolar system deviates from the classical bipolar–tetrapolar paradigm and, to our knowledge, has never been observed before. We propose a model for *MAT* evolution in the Basidiomycota in which the pseudo-bipolar system may represent a hitherto unforeseen gradual form of transition from an ancestral tetrapolar system to bipolarity.

## Author Summary

Sexual reproduction in fungi is regulated by genomic regions called *MAT* loci that determine sexual identity in a manner comparable to that of sex chromosomes in animal and plants. In most fungi, sexual reproduction is bipolar, i.e., two alternate and distinct sets of genes at the *MAT* locus determine two mating types (the equivalent to sexes). In the Basidiomycota, which is the fungal lineage that includes the mushrooms, a unique (tetrapolar) sexual reproduction system evolved in which the mating type is determined by two functionally independent and genetically unlinked regions. In addition, the tetrapolar system functions with multiple alleles for at least one the two classes of *MAT* genes. This potentially generates thousands of mating types, as observed for many mushroom species. Here we report on the molecular characterization of the mating system in the basidiomycetous red yeast *Sporidiobolus salmonicolor*, which belongs to the Pucciniomycotina, the earliest derived lineage of the Basidiomycota that has remained virtually unexplored with respect to gene content and structure of *MAT* loci. Our results revealed for the first time a mating system that is neither tetrapolar nor strictly bipolar, and we propose a model for the evolution of basidiomycete *MAT* loci that accommodates this novel finding.

## Introduction

In fungi, sexual reproduction systems have been well characterized at the molecular level in numerous species belonging to the Ascomycota and the Basidiomycota, revealing striking similarities with sex determining regions in animals and plants [1] along with unique features such as the occurrence of thousands of sexual identity (mating) types in a single species [2]. Most fungi capable of sexual reproduction are heterothallic [3] which means that mating occurs only between distinct haploid individuals with compatible mating types. However, homothallic (self-fertile) mating is not unusual and several different genetic mechanisms have been shown to form the basis for this sexual behaviour [4]–[7].

Sexual reproduction systems in fungi have served as invaluable eukaryotic models of development [8], transcriptional regulation [9], [10] and signalling pathways [11] but research in the field has also been spurred by the fact that for many plant or animal pathogens, sexual reproduction is intimately linked with virulence (e.g. the human pathogens *Cryptococcus neoformans* and *Candida albicans* [12] or the maize smut *Ustilago maydis* [13]).

The chromosomal regions that determine the mating type in fungi are called the *MAT* loci and vary extensively in length and in genetic content in different fungal lineages. In most species within the Ascomycota, the two *MAT* loci in sexually compatible haploid individuals are short and contain completely unrelated sequences (idiomorphs) encoding transcription factors that regulate post-mating sexual development. These transcription factors belong to the alpha-domain, homeodomain or HMG families [14]. In the phylum Basidiomycota, mating of compatible haploid partners – homokaryotic hyphae or haploid yeast cells – originates a dikaryotic filamentous stage where subsequently basidia and basidiospores (meiospores) are formed [15]. Pioneering work on the mushroom *Schizophyllum commune* provided the first glimpse of a unique sexual compatibility system, the so-called tetrapolar or bifactorial system [16]. This system is only found in basidiomycetes because, unlike all other fungi studied so far, they possess two independent molecular determinants of mating type [17]. Sexual compatibility is usually determined at a first level by lipopetide pheromones and plasma membrane pheromone receptors that mediate cell-cell recognition, with the exception of some homobasidiomycetes (the lineage that includes the mushrooms) where fusion of homokaryotic hyphae is not restrained by pheromone-mediated interactions [2], [18]. After cell fusion, progression through the sexual cycle requires overcoming a second compatibility hurdle that relies on a heterodimeric homeodomain transcription factor (HD1/HD2), encoded by a pair of divergently transcribed, closely linked genes [3], [19]–[21]. The HD1/HD2 heterodimer is a transcriptional regulator of post-mating sexual development that is only active in the dikaryon because dimerization is restricted to subunits that originate from genetically different individuals. Dimerization of HD1 and HD2 proteins encoded by the same gene pair is prevented in haploid individuals by a self/non-self recognition mechanism that has been well characterized in mushrooms [22]–[24] and in smut fungi [25], [26]. These studies showed that the self/non-self recognition domain resides on the highly variable N-terminal portion of the proteins. Accordingly, in HD1 proteins of the mushroom *Coprinus cinereus*, this domain was found to have exceptionally high evolution rates [27].

Almost without exception, the two classes of genes (encoding pheromone/pheromone receptors and HD1/HD2 transcription factors) are part of the *MAT* locus in basidiomycetes, which is the only genomic region for which the sequence varies according to the mating type. A notable aspect of the tetrapolar system is that it is multiallelic with respect to at least one of the two genetically unlinked classes of *MAT* genes, resulting in up to thousands of different mating types in some species [2], [3], [16], [28], [29]. Bipolar systems are, on the contrary, usually biallelic for both classes of genes [30], [31].

Tetrapolar species can be found in all three major lineages of Basidiomycota interspersed with bipolar species [16], [32] and, most significantly, do not seem to be uniformly distributed. In the subphylum Agaricomycotina, which encompasses the mushrooms, the majority of the species seems to be tetrapolar [16]. The subphylum Ustilaginomycotina harbours many plant pathogenic species like *U. maydis*, most of which exhibit tetrapolar mating systems very similar to those found in the Agaricomycotina. A substantial body of evidence [3], [30], [33] suggests that in these two subphyla, the few bipolar species were derived from tetrapolar ancestors as a result of coalescence between the regions encoding the two classes of *MAT* genes, like in the human pathogens *Cryptococcus neoformans* [30] and *Malassezia globosa* [34] and the barley smut *Ustilago hordei* [31]. In other cases (the mushrooms *Coprinellus disseminatus* and *Pholiota nameko*) the transition to bipolar behaviour was due to loss of the association of the pheromone receptor to the *MAT* locus [35], [36]. The third subphylum, the Pucciniomycotina, contains about one-third of all species of Basidiomycota [37]. The majority of the species in the Pucciniomycotina are rust fungi, a large group of economically important obligate plant parasites. Classical mating studies indicate a predominance of bipolar systems in this subphylum both for rusts [38], other plant parasites like *Microbotryum violaceum* [39] and for saprobic yeasts of the genera *Rhodosporidium* and *Sporidiobolus* [40], [41]. However, detailed molecular analyses elucidating *MAT* gene content and function are very limited except for *M. violaceum*, in which size dimorphic sex chromosomes were identified and preliminarily characterized [42] and the pheromone receptor genes of both mating types were recently identified [43]. Since molecular phylogenies and ultrastructure suggest that Pucciniomycotina diverged first among subphyla of Basidiomycota [44], the elucidation of the genetic structure of the mating systems in this lineage is of major importance for understanding the origin and evolution of basidiomycete *MAT* loci.

The lack of knowledge on the sexual reproduction mechanisms of the Pucciniomycotina is mainly a consequence of the obligate parasitism of the rust fungi that seriously constrains experimental manipulation. This problem can be circumvented by studying related saprophytic organisms like those of the genera *Rhodosporidium* and *Sporidiobolus* since they are capable of completing their life cycle in culture. This led us to the recent characterization of one of the pheromone receptor regions in several bipolar red yeast species, including the heterothallic red yeast *Sporidiobolus salmonicolor* [45].

Here we report on the molecular characterization of the mating system in *S. salmonicolor*. We identified, for the first time in the Pucciniomycotina, the divergently transcribed genes encoding the HD1 and HD2 transcription factors and we investigated how HD1/HD2 transcription factors and the pheromone receptor system interact to produce the bipolar mating behaviour observed in *S. salmonicolor*. A new, non-bipolar non-tetrapolar, sexual mechanism was unveiled, in which occasional disruptions of the genetic cohesion of the bipolar *MAT* locus originate new mating types in a process that parallels that of tetrapolar systems.

## Results/Discussion

### Identification of *MAT* genes in both mating types of *S. salmonicolor*

We recently identified *MAT* A1 (see the Methods section for changes in mating type designations in *S. salmonicolor*) pheromone receptor and pheromone precursor genes in the heterothallic bipolar red yeasts *Rhodosporidium toruloides* and *Sporidiobolus salmonicolor* and in *Sporobolomyces roseus* [45]. These studies failed to uncover HD1/HD2 transcription factor genes in the vicinity of the pheromone receptor in any of the species studied. However, HD1 and HD2 homologs [32], [45] were found in the completely sequenced genome of *Sporobolomyces roseus*, but at a large distance from the pheromone receptor gene. To get some insight in the genetic structure of *MAT* loci in red yeasts, we set out to characterize the *MAT* genotype of a set of 36 *S. salmonicolor* strains. This species was chosen because of previous studies concerning its mating behaviour [46] and of the availability of a 3× genome coverage in the form of Trace Archive sequences for one *MAT* A1 strain, considerably facilitating the identification of novel genes.

We first identified the pheromone receptor gene in the complementary mating type of *S. salmonicolor* (*MAT* A2). Degenerate primers based on the sequences available for homologous *MAT* A2 receptors, including that of the closely related *Rhodosporidium babjevae* for which Trace Archive genomic sequences are available, failed to amplify the *S. salmonicolor* gene. An alternative approach was conceived, based on our previous observation of a high degree of synteny between the same mating type in different red yeast species [45]. For this, the region surrounding the pheromone receptor gene in *R. babjevae* was assembled stepwise using Trace Archive sequences and was subsequently scrutinized for the presence of genes exhibiting a higher degree of conservation across species than pheromone receptor genes. Two putative genes encoding an LSm-like protein (*LSm7*) and a ribosomal protein L6 (*RibL6*) were found respectively upstream and downstream of *STE3.A2* in *R. babjevae*. Sequences homologous to these genes were readily found by BLASTN search in the *S. salmonicolor* Trace Archives but they were differently organized, since the sequenced strain (CBS 483) belongs to the opposite mating-type (*MAT* A1; Figure S1). PCR primers based on the *S. salmonicolor LSm7* and *RibL6* genes finally allowed the amplification of the intervening *S. salmonicolor STE3.A2* gene (GenBank accession number GU474641), attesting synteny conservation between *S. salmonicolor* and *R. babjevae* in this region within the same mating type. As expected, the predicted amino acid sequence of the *S. salmonicolor* receptor exhibited the highest similarity with the sequences of the *R. babjevae* (54%) and *M. violaceum* (47%) homologues.

The divergently transcribed genes encoding the HD1 and HD2 transcription factors were identified in the Trace Archives sequences of *S. salmonicolor* using the homologous sequences of *S. roseus*. Primers based on the homeodomain conserved region of these genes amplified a fragment encompassing the highly variable 5′ regions of both genes as well as the intergenic region between the *HD1* and *HD2* genes (Figure S1). Homology to the HD1 and HD2 proteins from *S. roseus* was, as expected, limited to the homeodomain region since all functional HD proteins characterized so far were found to have highly variable N-terminal domains, even when different *HD1/HD2* alleles from the same species are compared.

### Allele number and mating-type specificity of *HD1/HD2* and pheromone receptor genes in *S. salmonicolor*

Heterothallic red yeast species within the order Sporidiobolales were all described as having a bipolar behaviour in standard mating tests [40], [41]. Therefore, it was striking to notice that in *S. roseus*, the distance between the pheromone receptor and HD1/HD2 regions although still not accurately determined, was in any case larger than 800 Kb [32], [45].

**Figure 1:**
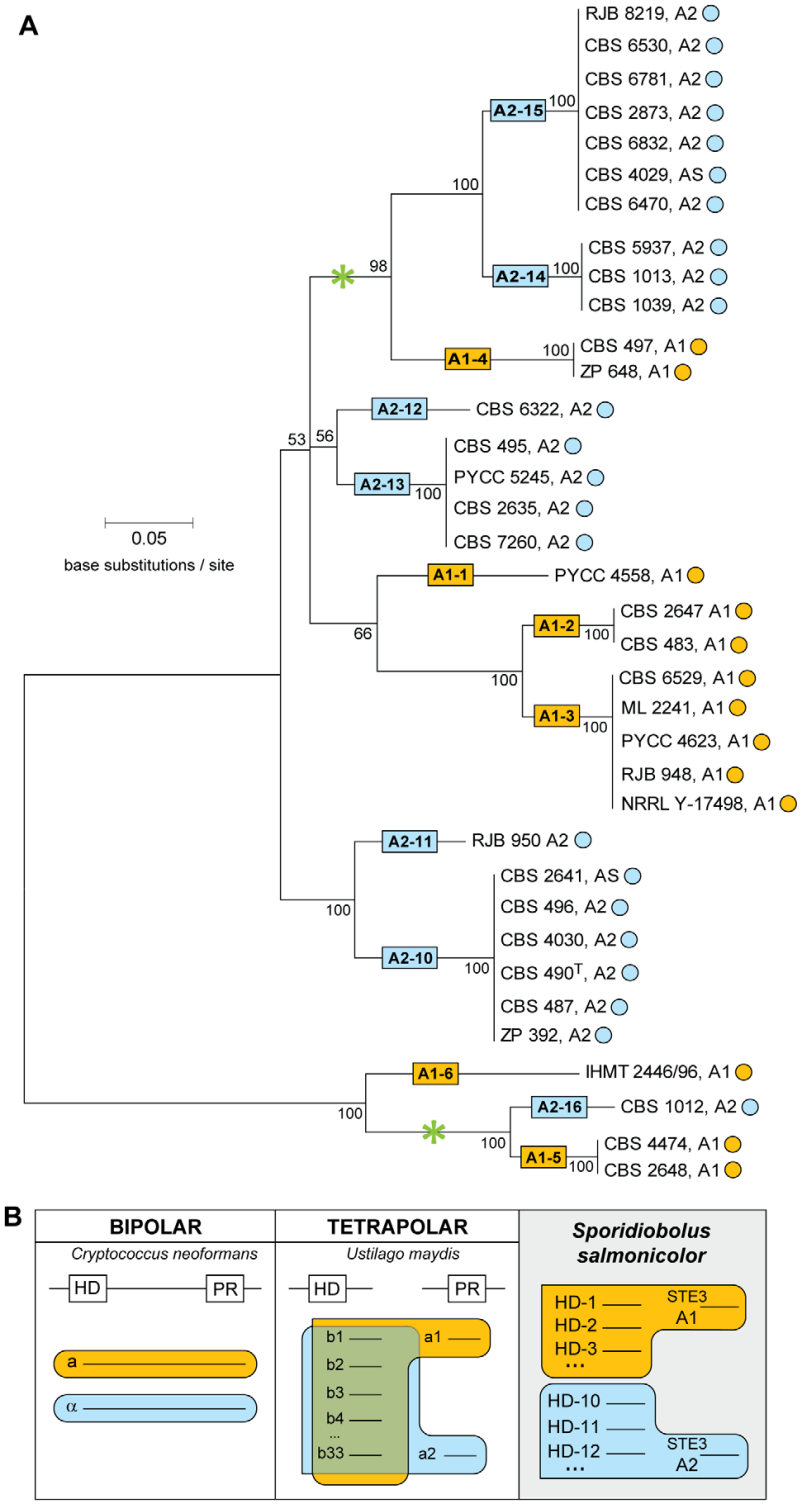
Diversity and phylogeny of *MAT* gene alleles in *S. salmonicolor*. (A) Phylogeny of *HD1/HD2* genes. Mating types A1, A2, and asexual strains are designated as A1, A2 and AS, respectively. Boxes designate the various *HD1/HD2* alleles with numerals after the mating type designation. Circles after strain numbers depict the type of pheromone receptor gene (yellow, *STE3.A1*; blue, *STE3.A2*). Asterisks indicate the two instances where common ancestry of *HD1/HD2* alleles associated with opposite mating types is best supported. (B) Comparison of the number and distribution of *MAT* gene alleles in emblematic species representing the different mating systems (HD, homeodomain region; PR, pheromone receptor region; the continuous or discontinuous line between the HD and PR boxes denotes genetic linkage or its absence, respectively).

Hence, if both classes of genes were part of a bipolar *MAT* locus with suppression of recombination over its entire length, it would be the largest such locus characterized so far in basidiomycetes. Such a region would nevertheless functionally resemble the *MAT* loci of *C. neoformans* [30], [47] and *U. hordei* [31], [48]. Alternatively, one of the compatibility check points might no longer be required to determine sexual identity, as previously observed for two mushroom species [35], [36]. In light of this, it was important to investigate how HD1/HD2 transcription factors and the pheromone receptor system interacted to produce the bipolar mating behavior. Isolation of both classes of *MAT* genes in *S. salmonicolor* enabled us to examine this question for the first time. We first used specific PCR primers to assess the correlation between the presence of the alternate pheromone receptor genes (*STE3.A1* and *STE.A2*) and mating behavior, in 36 natural isolates of *S. salmonicolor* (Table S1). Without exception, *STE3.A1* was present in *MAT* A1 strains, whereas the *STE3.A2* receptor gene was found in *MAT* A2 strains. Hence, the pheromone receptor was clearly associated to mating behaviour. Next, PCR fragments encoding the 5′ portions of the *HD1*and *HD2* genes were obtained from the 36 *S. salmonicolor* strains of both mating types and sequenced (GenBank accession numbers GU474649–GU474693). Surprisingly, instead of two *HD1/HD2* alleles, each strictly linked to one of the mating types, as might be expected for a bipolar species, 13 *HD1/HD2* alleles exhibiting substantial sequence divergence were uncovered (Figure 1A). Such numbers of *HD1/HD2* alleles were previously observed only for tetrapolar species [16]. In *U. maydis*, for example, 33 *HD1/HD2* alleles were identified [49] which do not have a particular association to either one of the two pheromone receptors because the two *MAT* regions are located on different chromosomes [3] and thus segregate independently at meiosis. On the contrary, in the bipolar species characterized so far, only two *HD1/HD2* alleles are present, each of which is linked to one of the two pheromone receptors forming two large bipolar loci [30], [31]. Recombination between the two genetically linked *MAT* regions is suppressed in these species as a result of extensive sequence divergence, gene inversions and the accumulation of repetitive elements reminiscent of sexual chromosomes in animals and some plants [1], [30], [31], [50]. In *S. salmonicolor*, each of the 13 alleles always appears associated with the same receptor, suggesting some form of genetic linkage between the two regions (Figure 1). However, each receptor is associated with seven (Ste3.A2) or six (Ste3.A1) different *HD1/HD2* alleles which is in sharp contrast with other bipolar species (Figure 1). There is apparently no bias regarding the geographic distribution of *HD1/HD2* alleles, since strains carrying the same *HD1/HD2* allele were isolated from very diverse locations worldwide (Table S1). To shed some light on the phylogenetic relationship between the *HD1/HD2* alleles, we also examined the alleles present in a very closely related species, *S. johnsonii*. Several lines of evidence suggest that *S. salmonicolor* and *S. johnsonii* are undergoing a speciation process in which pre-zygotic barriers are absent, since crosses between sexually compatible strains of the two species normally yield dikaryotic mycelium and teliospores [46]. Intriguingly, extant allele diversity seems to have been generated after the onset of this incipient speciation event, as *HD1/HD2* alleles from the two species are, in general, phylogenetically distinct (Figure S2). However, some incongruities between rDNA and *MAT* gene phylogenies can be observed (Figure S2), indicative of a certain degree of gene flow between the two species. For example, *S. salmonicolor* strain CBS 2634 has a *S. johnsonii* rather than a *S. salmonicolor* receptor allele and, in addition, a group of five *S. salmonicolor* strains carry *HD1/HD2* alleles (A1–5, A2–16, A1–6; Figure S2) which seem to be phylogenetically more related to the *S. johnsonii* clade. The intraspecific common ancestry of *HD1/HD2* alleles contrasts with the polymorphism observed for the pheromone receptor genes. The latter genes were found to exhibit a clear and ancient trans-specific polymorphism (Figure S3), as previously reported for *M. violaceum* [43].

**Figure 2:**
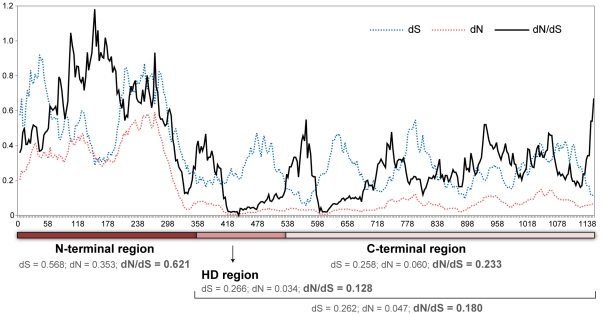
Evolution rate of the *HD1* gene in *S. salmonicolor*. Synonymous (dS), non-synonymous (dN) and dN/dS average values for all pairwise comparisons between nine alleles of the *HD1* gene. Y axis – evolution rates; X axis – base pairs of *HD1* coding region. Average values are given for the highly variable N-terminal domain which is involved in non-self recognition, and for the more conserved homeodomain (HD) and C-terminal domains.

Hence, the relationship between mating type and *HD1/HD2* allele number and distribution is unexpectedly complex in *S. salmonicolor*, and the possibility that the HD1/HD2 transcription factors might have recently lost their linkage to *MAT* and are no longer involved in determining sexual compatibility in this species could not be readily discarded. To examine this, we determined first the evolution rates (dN/dS) of nine *HD1* alleles of *S. salmonicolor* and *S. johnsonii* (GenBank accession numbers GU474694–GU474702). Prior studies in the mushroom *Coprinus cinereus* brought to light exceptionally high evolution rates for the domains in HD1 proteins involved in self/non-self recognition [27]. We observed even higher evolution rates for the homologous domains in the HD1 proteins of *S. salmonicolor* (>1 in some segments, indicative of adaptive selection; Figure 2 and Figure S4), which constituted the first evidence that these transcription factors contribute to determine sexual compatibility in *S. salmonicolor*, together with the pheromone receptor system.

### A pseudo-bipolar mating system

**Figure 3:**
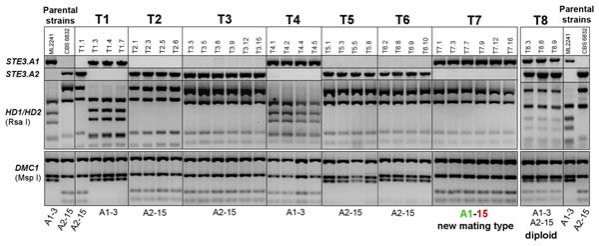
Segregation analysis of the *STE3* and *HD1/HD2* genes after meiosis. (A) T1 to T8 refer to 8 individually germinated teliospores obtained by micromanipulation from the cross between strains CBS 6832 (A2–15) and ML 2241 (A1–3). The result of the diagnostic PCRs for the presence of the alternate *STE3.A1* and *STE3.A2* genes and the identification of *HD1/HD2* amplicons by Rsa I digestion is shown. Screening of the parental origin of the *DMC1* allele by PCR and digestion with Msp I show clonality of the various colonies examined for each teliospore. For each germinated teliospore, three to six colonies were studied. With the exception of teliospore T1, only one mating type was recovered per meiosis presumably due to asynchronous germination of basidiospores (see Figure S5C). Teliospore T8 yielded a strain which is apparently diploid, and the new mating type was recovered from teliospore T7.

We sought further for an explanation of how the “one-to-many” ratio between pheromone receptor and *HD1/HD2* genes, characteristic of tetrapolar systems, results in bipolar behaviour with only two mating types in *S. salmonicolor*. To this end, we investigated the possibility that the cohesion of the bipolar *MAT* locus in *S. salmonicolor* might be occasionally disrupted, giving rise to new receptor/HD allele combinations. Such recombination events would explain the common ancestry of extant A1- and A2-linked *HD1/HD2* alleles (Figure 1A) and indeed the fact that all *HD1/HD2* alleles in the species seem to share a common ancestor more recent than the formation of the species itself (Figure S2). Therefore, bipolarity in *S. salmonicolor* could be a consequence of the scarcity of recombination events on the one hand and of the exceptionally high evolution rates of HD1/HD2 proteins on the other hand. The combination of these two factors would lead to the association of each extant *HD1/HD2* allele with only one of the alternate receptors, the hallmark of bipolarity. Alternatively, it could not be readily excluded that the observed apparent linkage could be due to population genetic phenomena related to the frequency of sexual reproduction and population size. To address the possibility of partial genetic linkage between the two *MAT* regions, we studied in detail the latter stages of the sexual cycle (germination of teliospores with the formation of basidia and basidiospores; Figure S5) in one cross between strains ML 2241 (*MAT* A1) and CBS 6832 (*MAT* A2). The progression through meiosis was microscopically monitored showing that although four nuclei could be observed after meiosis, only two basidiospores were formed, each arising from one of the two basidial compartments (Figure S5B). The two basidiospores were always found to be binucleate and they typically exhibited asynchronous germination (Figure S5). The molecular mating types of the progeny of eight meioses from the same cross were examined by micromanipulation of teliospores in the initial stages of germination. Several colonies isolated from each germinating teliospore were analysed and, in only one of the eight events examined (T1), were the two versions of the parental mating type genes recovered (Figure 3). Our interpretation is that the asynchronous germination of the two basidiospores results in a very strongly biased composition of the colony towards descendants of the basidiospore that germinates first. Micromanipulation of the basidiospores instead of the germinating teliospore would probably be required to recover the germination products of both basidiospores with higher frequency, but this proved to be technically very difficult due to the fact that germination takes place inside the agar and the structures to be manipulated are friable. Furthermore, the various colonies examined for each teliospore are most likely mitotic clones, as they invariably shared the same parental allele of the *DMC1* gene, (Figure 3, GenBank accession numbers HM133872–HM133874). In six of the meiotic events examined, linkage between the pheromone receptor and the HD1/HD2 regions was maintained, as would be expected in a bipolar system (Figure 3). Germination of one additional teliospore yielded a strain which was apparently diploid since it carries pheromone receptor and *HD1/HD2* alleles from both parental strains (Figure 3) and has a self-fertile phenotype with the formation of mycelium and teliospores. Self-fertile and asexual strains can be found among natural isolates of red yeasts of the Sporidiobolales [40], [41] and the origin of the first may be related to the occasional formation of diploid yeast strains. In addition to our present observations, the formation of diploid strains was also previously reported in *R. toruloides* [51]. However in none of the species studied so far are these diploid states prevailing, leading to the presumption that although these strains do not exhibit obvious growth defects, their genetic makeup may carry some selective disadvantages. Moreover, our preliminary observations suggest that teliospores formed by the *S. salmonicolor* diploid strain do not germinate, which argues against the co-existence of homothallic and heterothallic life styles in this species. Interestingly, the type strain of *S. johnsonii* (CBS 5470^T^) may have originated from a diploid strain because it carries both pheromone receptor alleles. However, only one *HD1/HD2* gene pair is present which may indicate that a self-compatible *HD1/HD2* gene pair was generated by recombination between the two parental alleles (Figure S2). The type strain of *S. johnsonii* is homothallic [41], [46] but does not form basidia, differing in that respect from all the other strains of this species, which carry only the *MAT* A1 receptor gene and are in general capable of mating with *S. salmonicolor MAT* A2 strains.

**Figure 4:**
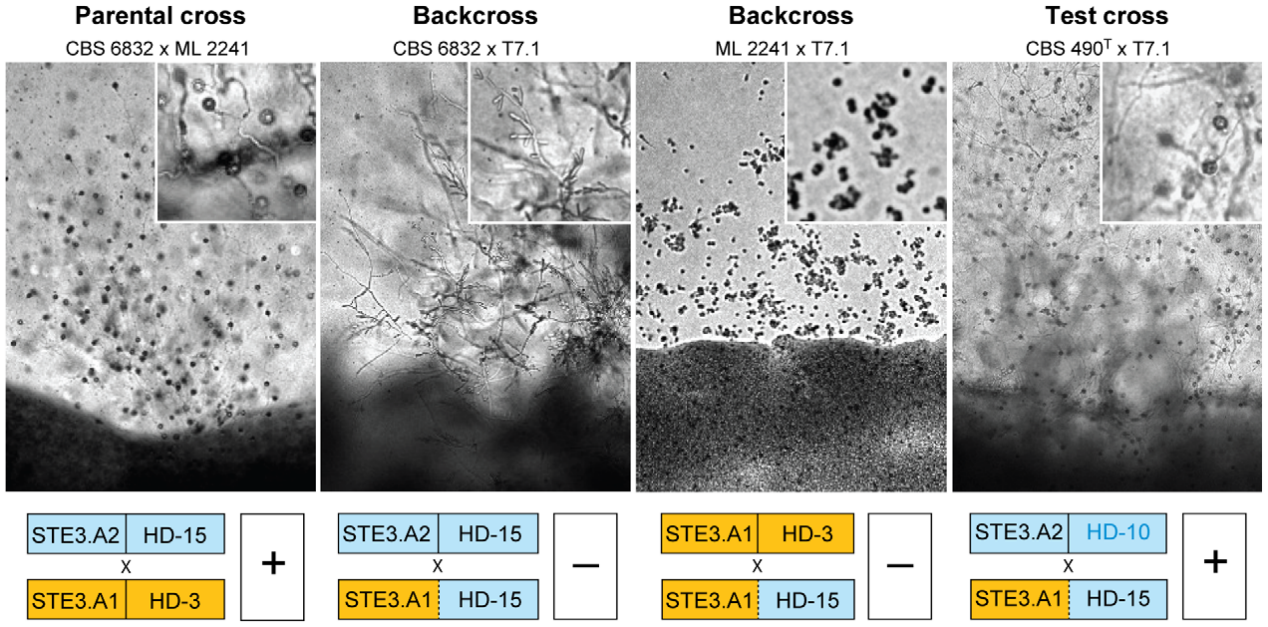
Sexual proficiency of T7.1, the new mating type of *S. salmonicolor*. For each cross the top section indicates the type of cross and the strains involved. Micrographs of crosses (4 days of incubation at 25°C on corn meal agar) are shown in the middle section (inserts depict details in higher magnification to show the presence or absence of teliospores). The bottom section shows the *STE3* and *HD* genotypes of each strain and the outcome of the cross evaluated by the presence (+) or absence (−) of mycelium with clamp connections and teliospores.

One germinated teliospore yielded the most striking result, since it produced an haploid strain in which recombination occurred, causing the *HD1/HD2* allele of parental strain CBS 6832 (*MAT* A2) to become associated with the Ste3.A1 receptor (Figure 3), a combination that is not found among natural isolates (Figure 1A). The new mating type (strain T7) was unable to complete the sexual cycle when crossed with either parental strain (Figure 4). A cross with the parent sharing the same pheromone receptor gene resulted in a complete failure to switch to filamentous growth, whereas homozygosity at the HD1/HD2 region precluded progression through the sexual cycle, but allowed some pseudohyphal growth, probably as a result of cell-cell pheromone signalling (Figure 4). This demonstrates unequivocally that both compatibility regions are required for sexual reproduction in *S. salmonicolor*. The new mating type did not show obvious defects in vegetative growth, exhibiting growth rates in synthetic and complete media similar to those of the parental strains (results not shown). It also had normal sexual proficiency when crossed with strains carrying different alleles at both *MAT* regions (Figure 4), forming dikaryotic mycelium with teliospores that germinate normally (result not shown).

**Figure 5:**
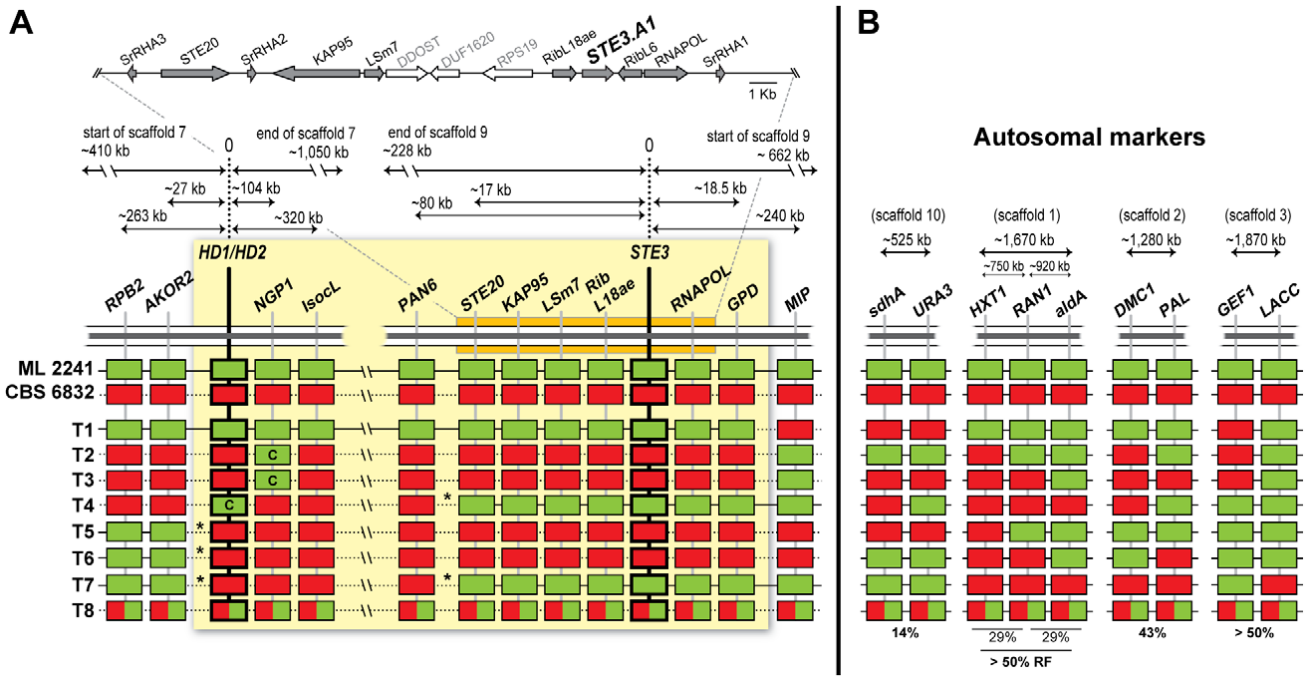
Analysis of meiotic progeny. (A) Genomic regions surrounding the *HD1/HD2* and pheromone receptor genes in *S. salmonicolor MAT* A1. Genes in the immediate vicinity of *STE3.A1* are depicted as block arrows to denote the direction of transcription. The same genes are highlighted with an orange box. Synteny with the homologous genomic region of *S. roseus* was confirmed for all genes except those shown as empty arrows (depicted in the arrangement present in *S. roseus*). Outside the highlighted region, genes present in the same *S. roseus* scaffold at different distances (indicated by the arrows) from *STE3.A1* were selected. Four genes were similarly selected from the *HD1/HD2* scaffold, assuming synteny with *S. roseus*. For eight independent teliospores (T1–T8) the parental origin of the genes is indicated. Genes from strain ML 2241 (A1–3) are shown in green and those from strain CBS 6832 (A2–15), in red. Asterisks mark sites were crossovers were detected. Possible gene conversion events are marked with “C”. The two scaffolds are depicted in the most likely orientation. The region shadowed in light yellow encompasses the putative pseudo-bipolar *MAT* locus in *S. salmonicolor MAT* A1. (B) Segregation patterns and recombination frequencies (RF) of nine autosomal markers located in four different scaffolds. The position and relative distances between the genes are depicted as in *S. roseus* genome. T8 is a diploid strain since it contains both parental alleles for all genes tested.

Recombination between the two classes of *MAT* genes seems to be possible but infrequent, a situation that, to our knowledge, has never been described before in basidiomycetes and can be regarded as intermediate between bipolar and tetrapolar systems. Therefore, this observation called for a closer examination of the meiotic events described above. In particular, it was important to establish how genetic markers unrelated to *MAT* segregated in these meiotic events, thereby getting some insight in the recombination frequency characteristic of this species in autosomal regions while simultaneously confirming that a normal meiosis had taken place in all the cases examined. It should be noted that a more precise determination of recombination frequencies and a statistically significant demonstration of linkage between genomic regions would require the examination of a larger number of meiotic products, but nevertheless examination of the eight available strains yields significant information concerning recombination inside and outside the *MAT* region. To characterize the haploid progeny strains, we chose nine genes located in four different *S. roseus* scaffolds (Figure 5). Pairs of genes located on the same scaffold were at distances ranging between 0.525 and 1.87 Mb from each other (Figure 5B). The selected genes were partially amplified in the two parental strains used in this cross (ML 2241 and CBS 6832) and sequence polymorphisms were scored which allowed the two parental alleles to be distinguished and their fate after meiosis to be tracked (GenBank accession numbers HM133857–HM133883). Sequencing of this set of genes in the seven haploid progeny strains confirmed that these markers segregate independently of *MAT*-specific genes (Figure 5). Markers located more than 1.2 Mb apart seem to be genetically unlinked, while the results suggested partial linkage for genes that are 525 to 920 kb apart, with an average of 32 kb/cM for these autosomal regions. The scaffolds containing the two *MAT* regions were subsequently subjected to a similar analysis. In scaffold 7, five genes, including the *HD1/HD2* pair were found to contain polymorphisms and were studied with respect to their parental origin in the seven haploid progeny strains (GenBank accession numbers GU474757–GU474780, HM133833–HM133856). In the scaffold harbouring the *STE3* gene, the parental origin of a total of eight additional genes exhibiting polymorphisms was determined (GenBank accession numbers GU474709–GU474756, HM133785–HM133832). The relative orientation of the two scaffolds could not be previously established [45], but the results of our scrutiny of meiotic recombination in these regions suggest linkage between the *IsocL* gene in scaffold 7 and the *PAN6* gene in scaffold 9. This implies that the relative orientation of the two scaffolds is most likely as depicted in Figure 5A. The analysis of the markers in these regions brought to light, in addition to sites where probably crossovers occurred, several instances where very likely gene conversion took place instead, one of which curiously involves the *HD1/HD2* gene pair (Figure 5A). In line with this, we found that the phylogeny of several genes located in the two scaffolds harbouring *MAT* genes also occasionally denotes signs of past gene conversion events when multiple strains of *S. salmonicolor* and *S. johnsonii* are examined (Figure S6). For example, the *LSm7* gene located close to *STE3*, exhibits an unusual mosaic phylogeny, which is species-specific in most of the gene but is *MAT* specific at the 3′-end, proximal to *STE3* (Figure S6). This suggests that this gene may have integrated the *MAT* locus long ago as might be expected from its close proximity to a key *MAT*-specific gene, but that the 5′end underwent conversion more recently, after incipient separation of the two species. Phylogenetic analysis of the *RibL18ae*, *RNAPOL*, *NGP1* and *AKOR2* genes also suggests gene conversion events in *S. salmonicolor* strain CBS 1012 (Figure S6). These observations support the idea that gene conversion is an important mechanism to maintain species-specific sequences at the *S. salmonicolor MAT* locus. This may, in turn, reflect the need to counteract the effect of recombination suppression which could lead to mating type-linked deleterious mutations. It is also important to note that sequence divergence between mating types of genes located in the vicinity of the receptor/pheromone *MAT* region is much lower in the red yeasts examined than, for example in *Cryptococcus neoformans* (Figure S6, [47]), although in the later species some events of gene conversion within the *MAT* loci have also been noticed [47].

Three crossovers were mapped to a region of 27 kb adjacent to the *HD1/HD2* gene pair (T5, T6 and T7; Figure 5), which seems to configure a hotspot for recombination (0.63 kb/cM) when compared with the average frequency of recombination observed for the autosomal regions examined (∼32 kb/cM). Interestingly, hotspots have been mapped close to the borders of the *MAT* locus in *Cryptococcus neoformans* [52] and were proposed to play an important role in the evolution of these specialized genomic regions. Three recombination events were also detected on the opposite side of the putative *MAT* locus in a region of ∼220 kb (T1, T2 and T3; Figure 5) but in this case only one genetic marker (*MIP*) was studied on one side of this position, which makes it difficult to distinguish between gene conversion and crossover events. Finally, two crossovers were detected in the proximity of the *PAN6* gene, one of which was the cause of the only instance of recombination detected between the *STE3* and the *HD1/HD2* genes (T7, Figure 5). In the other case (T4, Figure 5), the *MAT* A1 parental mating type prevailed, due to apparent gene conversion involving the *HD1/HD2* gene pair. In the 1.2 Mb region between the *HD1/HD2* and *PAN6* genes no crossovers seem to have occurred, suggesting that recombination frequency is lower than average or possibly suppressed in this region.

Hence, we propose that in *S. salmonicolor* a new type of fungal sex determining region operates, which is neither tetrapolar nor strictly bipolar. *S. salmonicolor* seems rather to have a large pseudo-bipolar *MAT* locus in which recombination in the region between the two *MAT* regions is infrequent but not suppressed, allowing for occasional disruptions of its genetic cohesion. The proposal of an intermediate system between bipolar and tetrapolar is based mainly on two kinds of observations, which are in line with each other. Firstly, the allelic distribution and number of *MAT* genes depicted in Figure 1 outlines a situation that is itself intermediate between bipolar and tetrapolar (multiple *HD1/HD2* alleles but only two mating types). Secondly, when trying to clarify this new finding, we found that recombination may occur in the intervening region between the two *MAT* regions, which provides a likely and plausible explanation for the observed resemblance with tetrapolar systems in what concerns evolution and number of *HD1/HD2* alleles. On the other hand, the genomic region around the receptor/pheromone locus exhibiting synteny breaks, gene inversions and sequence divergence between the two mating types is significantly larger in *S. salmonicolor* than in tetrapolar species with similar numbers of *MAT* gene alleles, like *U. maydis* (Figure S1, [47]). We envisage that the offspring of the relatively rare events of meiotic recombination in the *MAT* locus of *S. salmonicolor* can be rescued from reproductive isolation by the existence of multiple *HD1/HD2* alleles associated to each of the two alternate receptors in natural populations. In such a pseudo-bipolar system, negative frequency-dependent selection could operate to preserve *HD1/HD2* allele diversity and explain the exceptionally high evolution rates of HD1 proteins.

### A model for the evolution of *MAT* loci in basidiomycetes

Both the content and the organization of the basidiomycete *MAT* loci are unlike all other fungi, because compatibility is encoded in the *MAT* locus itself, while in other fungi the genes present in the *MAT* locus regulate expression of pheromones and pheromone receptors that are encoded elsewhere in the genome. The available data does not suffice to address definitively the question of how the basidiomycete *MAT* locus arose, but one hypothesis has been recently put forward that postulates that the pheromone receptor and/or the HD systems may have evolved in a self compatible manner [49]. The emergence of self-incompatible alleles by mutation or recombination could have subsequently laid the basis for the emergence of the tetrapolar system. There is currently insufficient evidence, also including our present observations, to state decisively whether the ancestral basidiomycete mating system was initially bipolar or tetrapolar, at the onset of the involvement of both the HD and pheromone receptor systems in determining mating specificity. However, our observations concerning the mating system in *S. salmonicolor*, suggest that novel and useful insights may be gained in this respect by a wider phylogenetic sampling of mating systems in the Pucciniomycotina.

**Figure 6:**
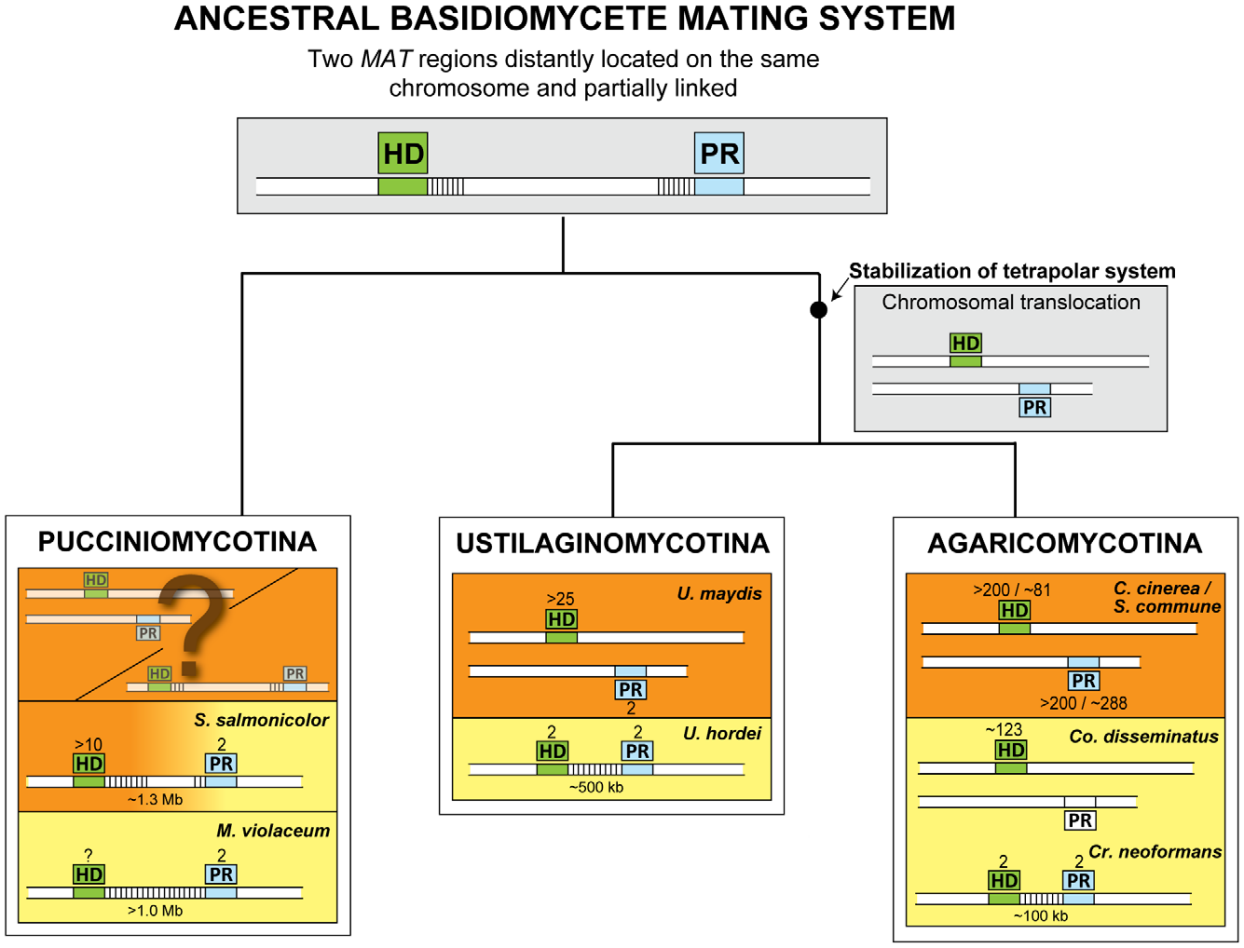
The pseudo-bipolar system and the evolution of *MAT* loci in Basidiomycota. The ancestral basidiomycete mating system may have been similar to the pseudo-bipolar system found in *S. salmonicolor*. The classic tetrapolar system emerged in the common ancestor of the Ustilaginomycotina and Agaricomycotina through a chromosomal translocation that placed the two *MAT* regions on different chromosomes. In *U. hordei* and *C. neoformans* a second translocation may have triggered a precipitous transition to bipolarity, as previously proposed [3], [33]. In the mushroom *Co. disseminatus*, the pheromone receptor (white box) ceased to be associated to *MAT* leading to bipolarity. Mating systems in the Pucciniomycotina may have evolved to extant systems exhibiting partial (*S. salmonicolor*) or complete (*M. violaceum*) suppression of recombination between the two *MA*T regions. Tetrapolar systems in this lineage might be present in *Leucosporidium scottii* and the rusts (Uredinales) but have not been characterized at the molecular level yet. In these systems, *MAT* regions may be distantly located on the same chromosome or on different chromosomes. The number of *MAT* gene alleles identified in each species is shown next to the boxes representing the homeodomain (HD) and pheromone receptor (PR) regions. Stripes between the HD and PR regions denote reduced or suppressed recombination.

In line with this, we subsequently looked into the *HD1/HD2* allelic distribution of *Rhodosporidium babjevae*, another bipolar red yeast species phylogenetically related to *S. salmonicolor*. Also in this case, multiple *HD1/HD2* alleles were associated with each receptor (Figure S7). Since this allelic distribution is the hallmark of the pseudo-bipolar system, we conclude that rather than being an oddity restricted to *S. salmonicolor*, this system is likely to operate in many of the yeast species of the order Sporidibolales [53] and probably also in other members of the subphylum Pucciniomycotina that were thus far thought to be bipolar. The most parsimonious explanation for this is, in our view, that a very similar system was present in the common ancestor of *Rhodosporidium* and *Sporidiobolus*. This implies that pseudo-bipolarity it is not a short-lived transitional mating system, like those presumed to have marked the transition to the bipolar state in *C. neoformans* [30], [31] and *U. hordei* [3]. Rather, it suggests that it may be evolving from an ancestral system in which the two classes of *MAT* genes were located on the same chromosome, but of which it is not possible to infer from currently available data whether the two *MAT* regions were genetically linked. The model shown in Figure 6 depicts the possibility that the ancestral basidiomycete mating system may have been similar to the pseudo-bipolar system found in *S. salmonicolor*. We highlighted this possibility among others, because the Pucciniomycotina is the earliest derived basidiomycete lineage and seems therefore more likely that members of this group have retained mating systems akin to the common ancestral system. In this scenario, the Pucciniomycotina would have diverged before the stabilization of the tetrapolar system (with the two *MAT* regions on different chromosomes), which is presumed to be the ancestor of the mating systems in the other two lineages (Ustilaginomycotina and Agaricomycotina) [49].

The model proposed in Figure 6 depicts this possibility and accommodates the possible occurrence of abrupt as well as gradual transitions to bipolar mating behaviour, the first but not the second being triggered by gross genomic rearrangements.

### Outlook

An intermediate, pseudo-bipolar mating system entails odds of inbreeding which are in between those observed for the bipolar and tetrapolar systems (50% and 25%, respectively; [33]). This may provide for some species the right balance between genetic stability (imparted by inbreeding) and variation (favoured by outcrossing) and may, in turn, delay the attainment of a strictly bipolar state if a system is drifting away from a tetrapolar configuration without abrupt occurrences, such as large chromosomal rearrangements. Our time course microscopic observation of meiotic events in *S. salmonicolor* indicates that basidiospores exhibit asynchronous germination (Figure S5C), decreasing the probability of a cross between siblings immediately after meiosis. This, together with the occasional meiotic recombination events, would generally decrease the odds of selfing when compared with a typical bipolar system, like that of the phylogenetically related plant pathogen *Microbotryum violaceum* [39]. Hence, the pseudo-bipolar mating system emerges as a remarkable novel context in which to explore how life-style, ecology and modes of reproduction interplay in the evolutionary history of eukaryotes.

## Methods

### Strains and mating-type designations

The list of strains studied and relevant information associated to them is given in Table S1. We re-assigned mating type designations of *S. salmonicolor* in order to match the “molecular mating type” identified by PCR detection of the pheromone receptor alleles (*STE3.A1 or STE3.A2*).

### Common settings for PCR amplification

Preparative PCR reactions were performed in a final volume of 50 µl with the following components (unless stated otherwise): 2 mM of MgCl_2_, 0.20 mM of each of the four deoxynucleoside triphosphates (GE Healthcare), 1% DMSO, 0.8 µM of each primer, 100 ng of genomic DNA, and 1 U Taq DNA polymerase (Fermentas, Canada). Thermal cycling consisted of a 5-minute denaturation step at 95°C, followed by 35 cycles of denaturation at 94°C for 30 s, 30 s at the annealing temperature (variable), and extension at 72°C (variable time). For annealing temperatures, extension times and primer sequences see Table S2. A final extension of 7 min at 72°C was performed at the end of each reaction.

### PCR amplification and sequencing of the *MAT A2* pheromone receptor (*STE3.A2* allele) in *S. salmonicolor*

The pheromone receptor gene (*pr-MatA2*) from *Microbotryum violaceum* (GenBank accession number EF584741) was used to perform a BLASTN search in the NCBI Trace Archive sequences of the genome project of *Rhodosporidium babjevae* strain WP1 (*MAT* A2; www.jgi.doe.gov/sequencing/statusreporter/psr.php?projectid=52130). The complete *STE3.A2* sequence of *R. babjevae*, in addition to the flanking regions, were assembled (Table S3) and subsequently scrutinized for the presence of genes exhibiting a higher degree of conservation across species than pheromone receptor genes. Sequences of two putative genes encoding an LSm-like protein (*LSm7*) and a ribosomal protein L6 (*RibL6*) were used to design a pair of degenerate primers (MC122 and MC123) to amplify and sequence the intervening region in *S. salmonicolor MAT* A2 strain CBS 490^T^. This region contained the *STE3.A2 S. salmonicolor* gene, which was sequenced by primer walking using primers MC126 and MC127 (Figure S1).

### PCR amplification and sequencing of the HD1/HD2 region in *S. salmonicolor*

Using the sequence of the *HD1* gene of *Sporobolomyces roseus* [45], a BLASTN search was performed in the NCBI Trace Archive database of *S. salmonicolor* and positive hits (Table S3) were assembled into a complete *HD1* gene. The upstream flanking region of the *HD1* was assembled stepwise and inspected using AUGUSTUS software [54], revealing the presence of a divergently transcribed *HD2* homologue. The deduced protein sequences of HD1 and HD2 of *S. roseus* and *S. salmonicolor* were aligned and the conserved regions were used to design specific primers (MC103 and MC104) to amplify and sequence the corresponding N-terminal and intergenic regions of the *HD1/HD2* genes in all *S. salmonicolor* and *S. johnsonii* strains (Figure S1, Table S3).

### Correlation between mating behaviour and the presence of pheromone receptor genes

To confirm mating behavior and sexual compatibility, 2–4 day-old cultures were crossed on corn meal agar (Difco), incubated at room temperature for 1 week, and examined microscopically using phase-contrast optics for production of mycelium with clamp connections and teliospores. Diagnostic PCR with primers for *STE3.A1* (MC053 and MC054) and *STE3.A2* (MC126 and MC127) were carried out to identify the pheromone receptor genes.

### Germination of teliospores and microscopic monitoring of meiosis in *S. salmonicolor*

Strains CBS 6832 (A2–6) and ML 2241 (A1–3) were mixed on corn meal agar (Difco) and incubated at room temperature (∼22°C) for 2 weeks, to allow for abundant production of teliospores. Small (∼0.5 cm) agar blocks containing teliospores were soaked in sterile distilled water for 8–10 weeks at 4°C. After this resting period, a suspension of teliospores was obtained by gently mashing the agar blocks with a small pestle. Teliospore germination was induced by transferring this suspension to 2% low melting point (LMP)-agarose plates incubated at room temperature for 2 weeks. To follow the germination of selected teliospores, small drops of the same suspension were transferred to 2% LMP-agarose-coated-slides. The different stages of meiosis were observed microscopically by staining several germinating teliospores with Safranin O [55]. Observations were made daily with 100× magnification using a Leica DMR microscope equipped with brightfield and differential interference contrast optics and microphotographs were recorded using a Leica DFC320 digital camera (Leica Microsystems GmbH, Wetzlar).

### Micromanipulation of teliospores and segregation analysis in *S. salmonicolor*

Teliospores resulting from the cross of strains CBS 6832 (A2–6) and ML 2241 (A1–3) were germinated as described above. Using a micromanipulator, teliospores in the initial stages of germination, i.e. with a non-septate basidium initial, were individually separated, transferred to MYP agar [46] and incubated at room temperature. The colony that formed after 1–2 days was re-streaked to obtain colonies derived from single cells. In three to six of these colonies, segregation of the two *MAT* regions was assessed by diagnostic PCR with specific primers for *STE3.A1* (MC053 and MC054), *STE3.A2* (MC126 and MC127) and *HD1/HD2* (MC103 and MC104). The *HD1/HD2* alleles were discriminated after amplification by digestion with restriction enzyme Rsa I.

### Sequence data and phylogenetic analyses

DNA and protein sequences were aligned with ClustalW 1.81 [56] with minor manual corrections. For the phylogeny of *HD1/HD2* alleles, FindModel (http://www.hiv.lanl.gov/content/sequence/findmodel/findmodel.html), which is an implementation of ModelTest [57], with the Akaike information criterion (AIC) was used. The Tamura-Nei (TrN)+G model [58], [59] (shape parameters 0.98049 and 1.08652 in Figure 1 and Figure S2 datasets, respectively) was employed in MEGA4 [60] using the Neighbor-Joining algorithm [61] and bootstrap values from 1000 replicates. For the phylogeny of the pheromone receptors (Figure S3) the substitution model of protein evolution was selected using ProtTest [62] with AIC. The WAG+I+G [59], [63], [64] model (shape parameter = 2.99; proportion of invariable sites = 0.035) was employed in PHYML [65] using maximum likelihood and bootstrap values from 100 replicates.

### Estimation of the evolution rates of the *HD1* gene

To obtain the sequence of the 3′end of *HD1* gene the region encompassing the homeodomain and a conserved motif located 10 bp upstream of the STOP codon was amplified using primers MC111 and MC112. The 5′end of the *HD1* gene was amplified using primers MC103 and MC104 (Figure S1). Evolution rates were estimated by a Window Analysis of dN and dS, using the online interface of WINA 0.34 [66], in a sliding window (size = 20) along the alignment of nine *HD1* alleles.

### Mating type specificity and phylogenetic analysis of genes located at variable distances from the HD1/HD2 and the pheromone receptor regions in *S. salmonicolor*

Based on the available genomic information of the closely related species *Sporobolomyces roseus*, and assuming that synteny is maintained in *S. salmonicolor* [41], eight genes from the pheromone receptor region (scaffold 9) and four genes from the HD1/HD2 region (scaffold 7) were selected. NCBI *S. salmonicolor* Trace Archive sequences of these genes were obtained (Table S3) and assembled. Primers were designed for partial sequencing of these genes (Table S2). Protein-coding DNA sequences were deduced after removal of putative introns, either manually or using AUGUSTUS software [54], automatically aligned using ClustalW and manually edited according to the superimposed amino acid sequences. Synonymous substitutions (dS) values and the divergence percentage between mating type-specific alleles were calculated using DnaSP 5.0 [67] for a set of 10 *S. salmonicolor* strains (Figure S6). Phylogenies were obtained in MEGA4 [60] using Maximum Parsimony (MP), with the close-neighbor interchange algorithm for heuristic searches and bootstrap values from 1000 replicates.

### Genotyping of the meiotic progeny

To detect differences among the meiotic progeny, we selected nine genes located on four different scaffolds (1, 2, 3 and 10; Figure 5) in the *S. roseus* genome. Sequences of these genes in *S. salmonicolor* were assembled from the NCBI Trace Archive (Table S3) and primers were designed to amplify partial sequences in parental strains CBS 6832 and ML 2241 (Table S2). Sequence polymorphisms were scored and used to track the parental origin of the alleles in all the meiotic progeny. This analysis was performed either by PCR and sequencing or by RFLP analysis (Table S2). The genes previously used to obtain mating type-specific phylogenies were similarly assessed in this genotyping analysis.

## Supporting Information

Figure S1 Organization of the genomic regions containing the pheromone receptor (*STE3*) and the *HD1/HD2* genes in *S. salmonicolor*. (A) Pheromone receptor region. Homologous genes are represented with the same colour. The pheromone genes (*SsRHA1*, *SsRHA2* and *SsRHA3*) are shown only in *MAT* A1 because their position in *MAT* A2 strains is currently unknown. Primers used to obtain the complete sequence of the *S. salmonicolor STE3.A2* gene are depicted by arrowheads. (B) The *HD1/HD2* region comprises two divergently transcribed homeodomain genes. The homeodomain motifs are indicated as black boxes and an intron present in the *HD2* gene is depicted. Black arrowheads indicate the position of the primers used to amplify the *HD1/HD2* region.
(0.43 MB TIF)

Figure S2 Phylogeny of *HD1/HD2* alleles in the species complex *S. salmonicolor*/*S. johnsonii*. Mating types A1, A2, and asexual strains are designated as A1, A2 and AS, respectively. Boxes designate the various *HD1/HD2* alleles with numerals after mating type designation. Yellow boxes correspond to *HD1/HD2* alleles associated to the pheromone receptor gene *STE3.A1* and blue boxes correspond to those associated with *STE3.A2*. Circles after strain numbers depict the type of pheromone receptor gene (yellow, *STE3.A1*; blue, *STE3.A2*). Half-coloured circles or boxes are used for *S. johnsonii* alleles. Lateral bars delimit *S. salmonicolor* (solid bar) and *S. johnsonii* (dashed bar) as defined by rDNA phylogeny [46]. The type strain of *S. johnsonii* (self-fertile) possesses both receptor genes *STE3.A1* and *STE3.A2*. Asterisks indicate the two instances where (post-speciation) common ancestry of *HD1/HD2* alleles associated with opposite mating types is best supported.
(0.86 MB TIF)

Figure S3 Phylogeny of pheromone receptors in the Basidiomycota. (A) Phylogeny and estimated time of divergence based on that of Devier *et al.* [43]; 450 MY, represents the divergence between ascomycetes and basidiomycetes and 370 MY, corresponds to the divergence between the pheromone receptor alleles in *Microbotryum violaceum*. The phylogeny was estimated by PHYML 3.0 using representative species of the major lineages in the Basidiomycota. Numbers on branches represent statistical support using 100 bootstraps replicates (see Methods for details). Coloured circles depict phylogenetic groups as defined in the rDNA phylogeny [15] shown in (B). Sequence accession numbers are indicated in brackets after species names.
(0.74 MB TIF)

Figure S4 Alignment of the HD1 proteins of *S. salmonicolor* and *S. johnsonii*. Alignment of the nine deduced protein sequences used in the evolutionary rates analysis (dN/dS). Coloured bars indicate the same three domains of the HD1 protein as in Figure 2. Sequences marked with an asterisk belong to the sibling species *S. johnsonii*.
(0.86 MB TIF)

Figure S5 Life cycle of *Sporidiobolus salmonicolor*. (A) Diagrammatic representation of the saprobic life cycle of *S. salmonicolor*. (B) Different stages of teliospore germination and formation of basidia and basidiospores; nuclei were stained with safranin O and are indicated by arrows: (i) initial stage of teliospore [Ts] germination with diploid nucleus and basidium initial [Ba]; (ii) migration of the diploid nucleus to the immature basidium; (iii) first meiotic division; (iv) formation of septum [Sp] originating a two-celled basidium; (v) second meiotic division yielding four nuclei; (vi) formation of two large binucleated basidiospores [Bs]; (vii) haploid uninucleated vegetative yeast cells [vc] resulting from basidiospore germination. Bars = 2.5 µm. (C) Time course observation of asynchronous germination of the basidiospores (i–iii). Note that, contrary to the basidiospore on the left, the basidiospore on the right formed yeast cells. Bars = 5 µm.
(5.94 MB TIF)

Figure S6 Phylogeny of several genes located in the two scaffolds harbouring *MAT* genes. The phylograms represent the single most likely tree for the selected genes located in the *STE3 region* (A) and in the *HD1/HD2* region (B). Trees were generated under the close-neighbor interchange algorithm in a heuristic search. Numbers on branches indicate statistical support calculated from 1000 bootstrap replicates. Possible gene conversion events are marked with an asterisk after the strain number. For the majority of the genes, sequences of strains of *MAT A2* (indicated by blue circles) are highly similar. In contrast, *MAT* A1 strains exhibit more sequence divergence, suggesting the existence of two lineages within this mating type (strains indicated by yellow and orange circles). Half-colored circles indicate *S. johnsonii* strains. Colored-shaded trees depict genes with sequence divergence clearly associated with mating type in *S. salmonicolor*. The position of the *LSm7* gene (light pink) relative to the *STE3* gene is shown for both mating types. Values for synonymous substitutions (dS) and divergence percentage (Dxy, %) are given for all genes (except for *STE3*) and in each pair comparisons.
(1.39 MB TIF)

Figure S7 Phylogeny of *HD1/HD2* alleles in *Rhodosporidium babjevae*. The pheromone receptor gene (*STE3.A1*, yellow circles; *STE3.A2*, blue circles) and sequence accession numbers (in brackets) are indicated after strain number. The tree was reconstructed with Neighbour-joining and TrN+G model (shape parameter = 1.68453). Bootstrap values (>50%) from 1000 replicates are shown. *S. salmonicolor* and *S. johnsonii* are included for comparison.
(0.37 MB TIF)

Table S1 List of *S. salmonicolor* and *S. johnsonii* strains used in this study and relevant information pertaining to them.
(0.73 MB PDF)

Table S2 List of primers, specific PCR conditions, and restriction enzymes used for the RFLP analysis.
(0.50 MB PDF)

Table S3 NCBI Trace Archives sequences used in this study.
(0.73 MB PDF)

## References

[1] Fraser JA, Heitman J (2005). Chromosomal sex-determining regions in animals, plants and fungi.. Curr Opin Genet Dev.

[2] Casselton LA, Olesnicky NS (1998). Molecular genetics of mating recognition in basidiomycete fungi.. Microbiol Mol Biol Rev.

[3] Bakkeren G, Kronstad JW (1994). Linkage of mating-type loci distinguishes bipolar from tetrapolar mating in basidiomycetous smut fungi.. Proc Natl Acad Sci U S A.

[4] Hicks JB, Herskowitz I (1976). Interconversion of yeast mating types I. Direct observations of the action of the homothallism (*HO*) gene.. Genetics.

[5] Rodriguez-Carres M, Findley K, Sun S, Dietrich FS, Heitman J (2010). Morphological and genomic characterization of *Filobasidiella depauperata*: a homothallic sibling species of the pathogenic *Cryptococcus* species complex.. PLoS ONE.

[6] Lin X, Hull CM, Heitman J (2005). Sexual reproduction between partners of the same mating type in *Cryptococcus neoformans*.. Nature.

[7] Alby K, Schaefer D, Bennett RJ (2009). Homothallic and heterothallic mating in the opportunistic pathogen *Candida albicans*.. Nature.

[8] Zarnack K, Feldbrügge M (2007). mRNA trafficking in fungi.. Mol Genet Genomics.

[9] Gustin MC, Albertyn J, Alexander M, Davenport K (1998). MAP kinase pathways in the yeast *Saccharomyces cerevisiae*.. Microbiol Mol Biol Rev.

[10] Basse CW, Farfsing JW (2006). Promoters and their regulation in *Ustilago maydis* and other phytopathogenic fungi.. FEMS Microbiol Lett.

[11] Xue C, Hsueh YP, Heitman J (2008). Magnificent seven: roles of G protein-coupled receptors in extracellular sensing in fungi.. FEMS Microbiol Rev.

[12] Nielsen K, Heitman J (2007). Sex and virulence of human pathogenic fungi.. Adv Genet.

[13] Bakkeren G, Kämper J, Schirawski J (2008). Sex in smut fungi: Structure, function and evolution of mating-type complexes.. Fungal Genet Biol.

[14] Butler G, Heitman J, Kronstad JW, Taylor JW, Casselton LA (2007). The evolution of *MAT*: the Ascomycetes.. Sex in Fungi: Molecular Determination and Evolutionary Implications.

[15] Hibbett DS, Binder M, Bischoff JF, Blackwell M, Cannon PF (2007). A higher-level phylogenetic classification of the Fungi.. Mycol Res.

[16] Raper JR (1966). Genetics of Sexuality in Higher Fungi.

[17] Kronstad JW, Staben C (1997). Mating type in filamentous fungi.. Annu Rev Genet.

[18] Kamada T (2002). Molecular genetics of sexual development in the mushroom *Coprinus cinereus*.. Bioessays.

[19] Kronstad JW, Leong SA (1989). Isolation of two alleles of the *b* locus of *Ustilago maydis*.. Proc Natl Acad Sci U S A.

[20] Kües U, Richardson WV, Tymon AM, Mutasa ES, Göttgens B (1992). The combination of dissimilar alleles of the *Aα* and *Aβ* gene complexes, whose proteins contain homeo domain motifs, determines sexual development in the mushroom *Coprinus cinereus*.. Genes Dev.

[21] Stankis MM, Specht CA, Yang H, Giasson L, Ullrich RC (1992). The *Aα* mating locus of *Schizophyllum commune* encodes two dissimilar multiallelic homeodomain proteins.. Proc Natl Acad Sci U S A.

[22] Kües U, Asante-Owusu RN, Mutasa ES, Tymon AM, Pardo EH (1994). Two classes of homeodomain proteins specify the multiple *A* mating types of the mushroom *Coprinus cinereus*.. Plant Cell.

[23] Banham AH, Asante-Owusu RN, Gottgens B, Thompson S, Kingsnorth CS (1995). An N-terminal dimerization domain permits homeodomain proteins to choose compatible partners and initiate sexual development in the mushroom *Coprinus cinereus*.. Plant Cell.

[24] Spit A, Hyland RH, Mellor EJ, Casselton LA (1998). A role for heterodimerization in nuclear localization of a homeodomain protein.. Proc Natl Acad Sci U S A.

[25] Gillissen B, Bergemann J, Sandmann C, Schroeer B, Bölker M (1992). A two-component regulatory system for self/non-self recognition in *Ustilago maydis*.. Cell.

[26] Kämper J, Reichmann M, Romeis T, Bölker M, Kahmann R (1995). Multiallelic recognition: nonself-dependent dimerization of the bE and bW homeodomain proteins in *Ustilago maydis*.. Cell.

[27] Badrane H, May G (1999). The divergence-homogenization duality in the evolution of the *b1* mating type gene of *Coprinus cinereus*.. Mol Biol Evol.

[28] Kothe E (1996). Tetrapolar fungal mating types: sexes by the thousands.. FEMS Microbiol Rev.

[29] Hsueh YP, Heitman J (2008). Orchestration of sexual reproduction and virulence by the fungal mating-type locus.. Curr Opin Microbiol.

[30] Lengeler KB, Fox DS, Fraser JA, Allen A, Forrester K (2002). Mating-type locus of *Cryptococcus neoformans*: a step in the evolution of sex chromosomes.. Eukaryot Cell.

[31] Lee N, Bakkeren G, Wong K, Sherwood JE, Kronstad JW (1999). The mating-type and pathogenicity locus of the fungus *Ustilago hordei* spans a 500-kb region.. Proc Natl Acad Sci U S A.

[32] Morrow CA, Fraser JA (2009). Sexual reproduction and dimorphism in the pathogenic basidiomycetes.. FEMS Yeast Res.

[33] Hsueh YP, Fraser JA, Heitman J (2008). Transition in sexuality: recapitulation of an ancestral tri- and tetrapolar mating system in *Cryptococcus neoformans*.. Eukaryot Cell.

[34] Xu J, Saunders CW, Hu P, Grant RA, Boekhout T (2007). Dandruff-associated *Malassezia* genomes reveal convergent and divergent virulence traits shared with plant and human fungal pathogens.. Proc Natl Acad Sci U S A.

[35] James TY, Srivilai P, Kües U, Vilgalys R (2006). Evolution of the bipolar mating system of the mushroom *Coprinellus disseminatus* from its tetrapolar ancestors involves loss of mating-type-specific pheromone receptor function.. Genetics.

[36] Yi R, Tachikawa T, Ishikawa M, Mukaiyama H, Bao D (2009). Genomic structure of the *A* mating-type locus in a bipolar basidiomycete, *Pholiota nameko*.. Mycol Res.

[37] Swann EC, Frieders EM, McLaughlin DJ, Mclaughlin DJ, McLaughlin EG, Lemke PA (2001). Urediniomycetes.. The Mycota VII. Systematics and Evolution. Part B.

[38] Buller AHR (1950). Researches on Fungi. Volume VII. The sexual process in the Uredinales.

[39] Giraud T, Yockteng R, López-Villavicencio M, Refrégier G, Hood ME (2008). Mating system of the anther smut fungus *Microbotryum violaceum*: selfing under heterothallism.. Eukaryot Cell.

[40] Sampaio JP, Kurtzman CP, Fell JW, Boekhout T (2010). *Rhodosporidium* Banno.. The Yeasts, a taxonomic study, 5th ed.

[41] Sampaio JP, Kurtzman CP, Fell JW, Boekhout T (2010). *Sporidiobolus* Nyland.. The Yeasts, a taxonomic study, 5th ed.

[42] Hood ME (2002). Dimorphic mating-type chromosomes in the fungus *Microbotryum violaceum*.. Genetics.

[43] Devier B, Aguileta G, Hood ME, Giraud T (2009). Ancient trans-specific polymorphism at pheromone receptor genes in basidiomycetes.. Genetics.

[44] Stajich JE, Berbee ML, Blackwell M, Hibbett DS, James TY (2009). The Fungi.. Curr Biol.

[45] Coelho MA, Rosa A, Rodrigues N, Fonseca A, Gonçalves P (2008). Identification of mating type genes in the bipolar basidiomycetous yeast *Rhodosporidium toruloides*: first insight into the *MAT* locus structure of the *Sporidiobolales*.. Eukaryot Cell.

[46] Valério E, Gadanho M, Sampaio JP (2008). *Sporidiobolus johnsonii* and *Sporidiobolus salmonicolor* revisited.. Mycol Prog.

[47] Fraser JA, Diezmann S, Subaran RL, Allen A, Lengeler KB (2004). Convergent evolution of chromosomal sex-determining regions in the animal and fungal kingdoms.. PLoS Biol.

[48] Bakkeren G, Jiang G, Warren RL, Butterfield Y, Shin H (2006). Mating factor linkage and genome evolution in basidiomycetous pathogens of cereals.. Fungal Genet Biol.

[49] Fraser JA, Hsueh YP, Findley KM, Heitman J, Heitman J, Kronstad JW, Taylor JW, Casselton LA (2007). Evolution of the mating-type locus: the basidiomycetes.. Sex in Fungi: Molecular Determination and Evolutionary Implications.

[50] Fraser JA, Heitman J (2004). Evolution of fungal sex chromosomes.. Mol Microbiol.

[51] Abe K, Sasakuma T (1986). Identification of a diploid self-sporulating cycle in the basidiomycetous yeast *Rhodosporidium toruloides*.. J Gen Microbiol.

[52] Hsueh Y-P, Idnurm A, Heitman J (2006). Recombination hotspots flank the *Cryptococcus* mating-type locus: implications for the evolution of a fungal sex chromosome.. PLoS Genet.

[53] Sampaio JP, Gadanho M, Bauer R, Weiss M (2003). Taxonomic studies in the Microbotryomycetidae: *Leucosporidium golubevii* sp. nov., *Leucosporidiella* gen. nov. and the new orders Leucosporidiales and Sporidiobolales.. Mycol Prog.

[54] Stanke M, Diekhans M, Baertsch R, Haussler D (2008). Using native and syntenically mapped cDNA alignments to improve *de novo* gene finding.. Bioinformatics.

[55] Dhingra OD, Sinclair JB (1995). Basic Plant Pathology Methods 2d ed.

[56] Thompson JD, Higgins DG, Gibson TJ (1994). CLUSTAL W: Improving the sensitivity of progressive multiple sequence alignment through sequence weighting, position-specific gap penalties and weight matrix choice.. Nucleic Acids Res.

[57] Posada D, Crandall KA (1998). MODELTEST: testing the model of DNA substitution.. Bioinformatics.

[58] Tamura K, Nei M (1993). Estimation of the number of nucleotide substitutions in the control region of mitochondrial DNA in humans and chimpanzees.. Mol Biol Evol.

[59] Yang Z (1994). Maximum likelihood phylogenetic estimation from DNA sequences with variable rates over sites: approximate methods.. J Mol Evol.

[60] Tamura K, Dudley J, Nei M, Kumar S (2007). MEGA4: Molecular Evolutionary Genetics Analysis (MEGA) software version 4.0.. Mol Biol Evol.

[61] Saitou N, Nei M (1987). The neighbor-joining method: a new method for reconstructing phylogenetic trees.. Mol Biol Evol.

[62] Abascal F, Zardoya R, Posada D (2005). ProtTest: Selection of best-fit models of protein evolution.. Bioinformatics.

[63] Whelan S, Goldman NA (2001). A general empirical model of protein evolution derived from multiple protein families using a maximum-likelihood approach.. Mol Biol Evol.

[64] Reeves JH (1992). Heterogeneity in the substitution process of amino acid sites of proteins coded for by mitochondrial DNA.. J Mol Evol.

[65] Guindon S, Gascuel O (2003). A simple, fast and accurate algorithm to estimate large phylogenies by maximum likelihood.. Syst Biol.

[66] Endo T, Ikeo K, Gojobori T (1996). Large-scale search for genes on which positive selection may operate.. Mol Biol Evol.

[67] Librado P, Rozas J (2009). DnaSP v5: A software for comprehensive analysis of DNA polymorphism data.. Bioinformatics.

