# Supplementary figures and images for: A Deviation from the Bipolar-Tetrapolar Mating Paradigm in an Early Diverged Basidiomycete
Source: PLoS Genet. 2010 Aug 5;6(8):e1001052. doi: 10.1371/journal.pgen.1001052 (PMC2916851; doi:10.1371/journal.pgen.1001052)

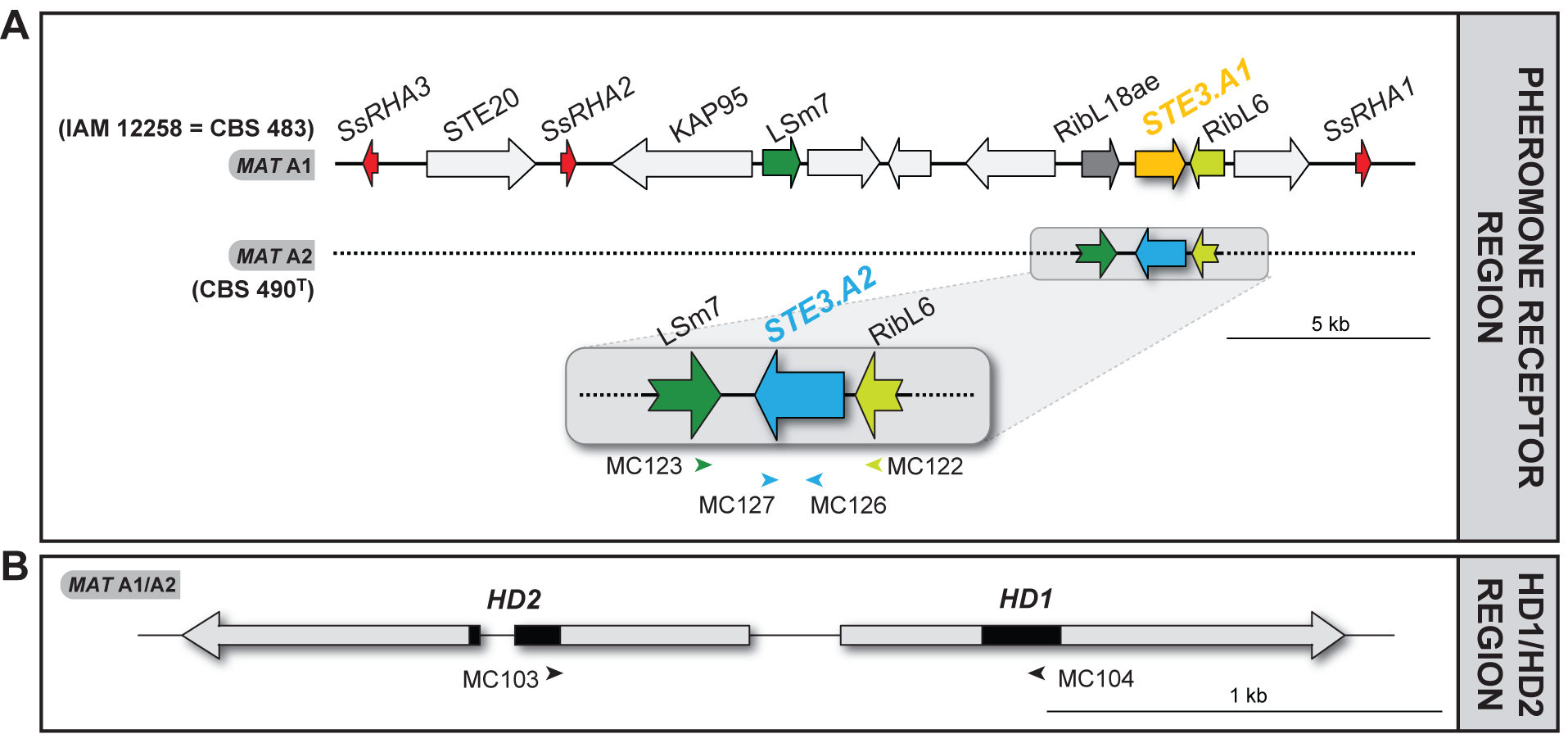

Supplement: Figure S1 — Organization of the genomic regions containing the pheromone receptor (STE3) and the HD1/HD2 genes in S. salmonicolor. (A) Pheromone receptor region. Homologous genes are represented with the same colour. The pheromone genes (SsRHA1, SsRHA2 and SsRHA3) are shown only in MAT A1 because their position in MAT A2 strains is currently unknown. Primers used to obtain the complete sequence of the S. salmonicolor STE3.A2 gene are depicted by arrowheads. (B) The HD1/HD2 region comprises two divergently transcribed homeodomain genes. The homeodomain motifs are indicated as black boxes and an intron present in the HD2 gene is depicted. Black arrowheads indicate the position of the primers used to amplify the HD1/HD2 region. (0.43 MB TIF) [file pgen.1001052.s001.tif]

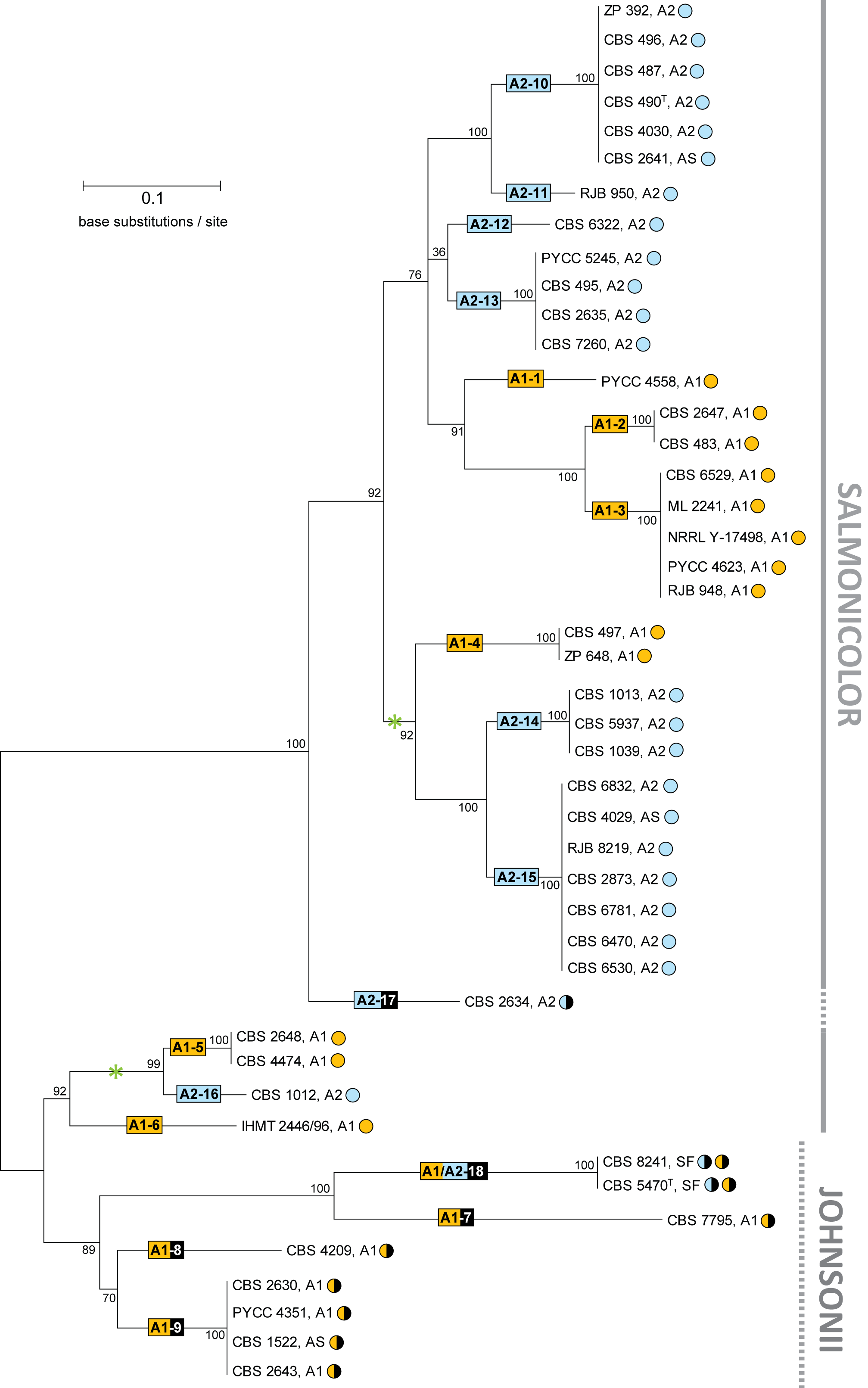

Supplement: Figure S2 — Phylogeny of HD1/HD2 alleles in the species complex S. salmonicolor/S. johnsonii. Mating types A1, A2, and asexual strains are designated as A1, A2 and AS, respectively. Boxes designate the various HD1/HD2 alleles with numerals after mating type designation. Yellow boxes correspond to HD1/HD2 alleles associated to the pheromone receptor gene STE3.A1 and blue boxes correspond to those associated with STE3.A2. Circles after strain numbers depict the type of pheromone receptor gene (yellow, STE3.A1; blue, STE3.A2). Half-coloured circles or boxes are used for S. johnsonii alleles. Lateral bars delimit S. salmonicolor (solid bar) and S. johnsonii (dashed bar) as defined by rDNA phylogeny [46]. The type strain of S. johnsonii (self-fertile) possesses both receptor genes STE3.A1 and STE3.A2. Asterisks indicate the two instances where (post-speciation) common ancestry of HD1/HD2 alleles associated with opposite mating types is best supported. (0.86 MB TIF) [file pgen.1001052.s002.tif]

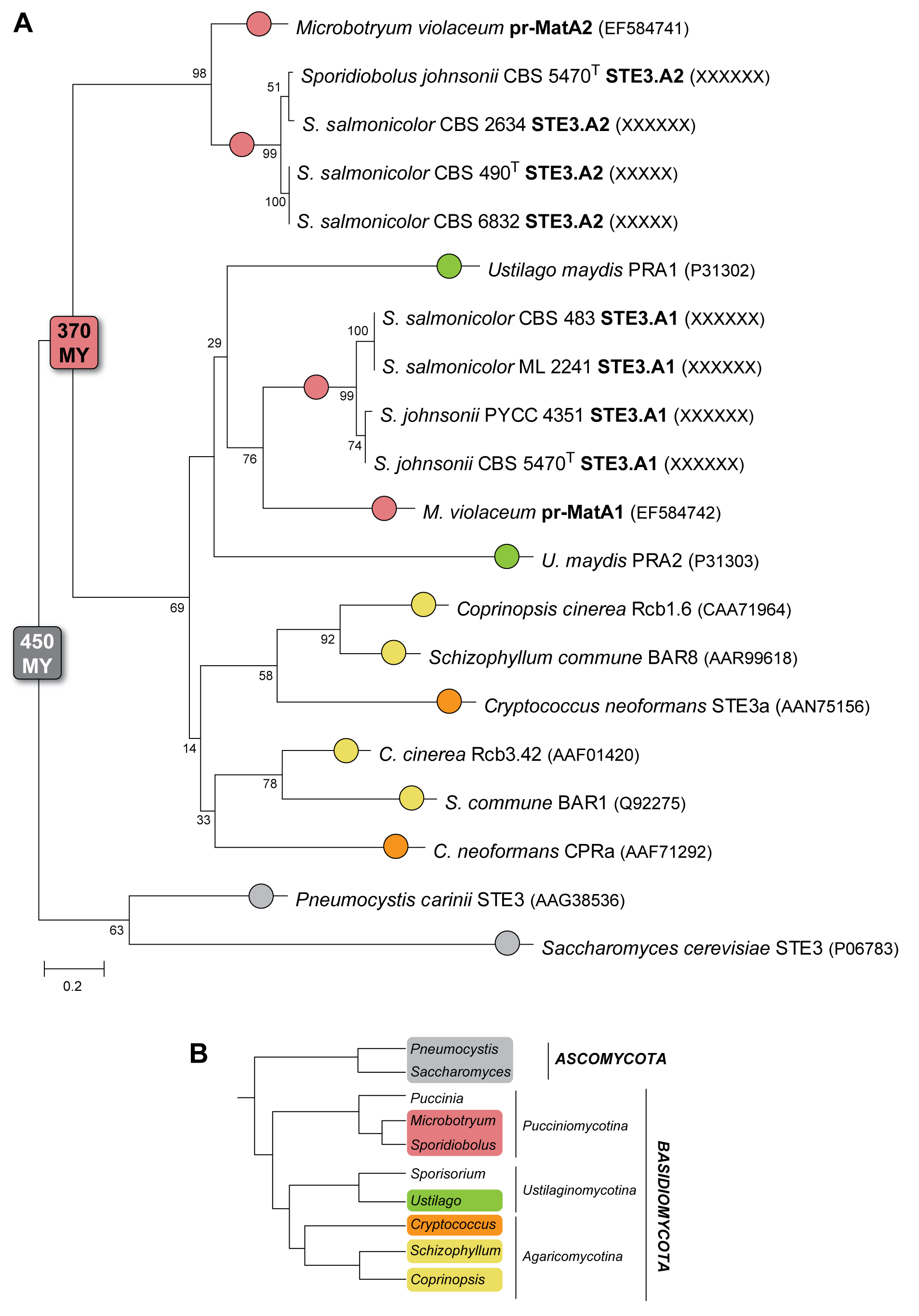

Supplement: Figure S3 — Phylogeny of pheromone receptors in the Basidiomycota. (A) Phylogeny and estimated time of divergence based on that of Devier et al. [43]; 450 MY, represents the divergence between ascomycetes and basidiomycetes and 370 MY, corresponds to the divergence between the pheromone receptor alleles in Microbotryum violaceum. The phylogeny was estimated by PHYML 3.0 using representative species of the major lineages in the Basidiomycota. Numbers on branches represent statistical support using 100 bootstraps replicates (see Methods for details). Coloured circles depict phylogenetic groups as defined in the rDNA phylogeny [15] shown in (B). Sequence accession numbers are indicated in brackets after species names. (0.74 MB TIF) [file pgen.1001052.s003.tif]

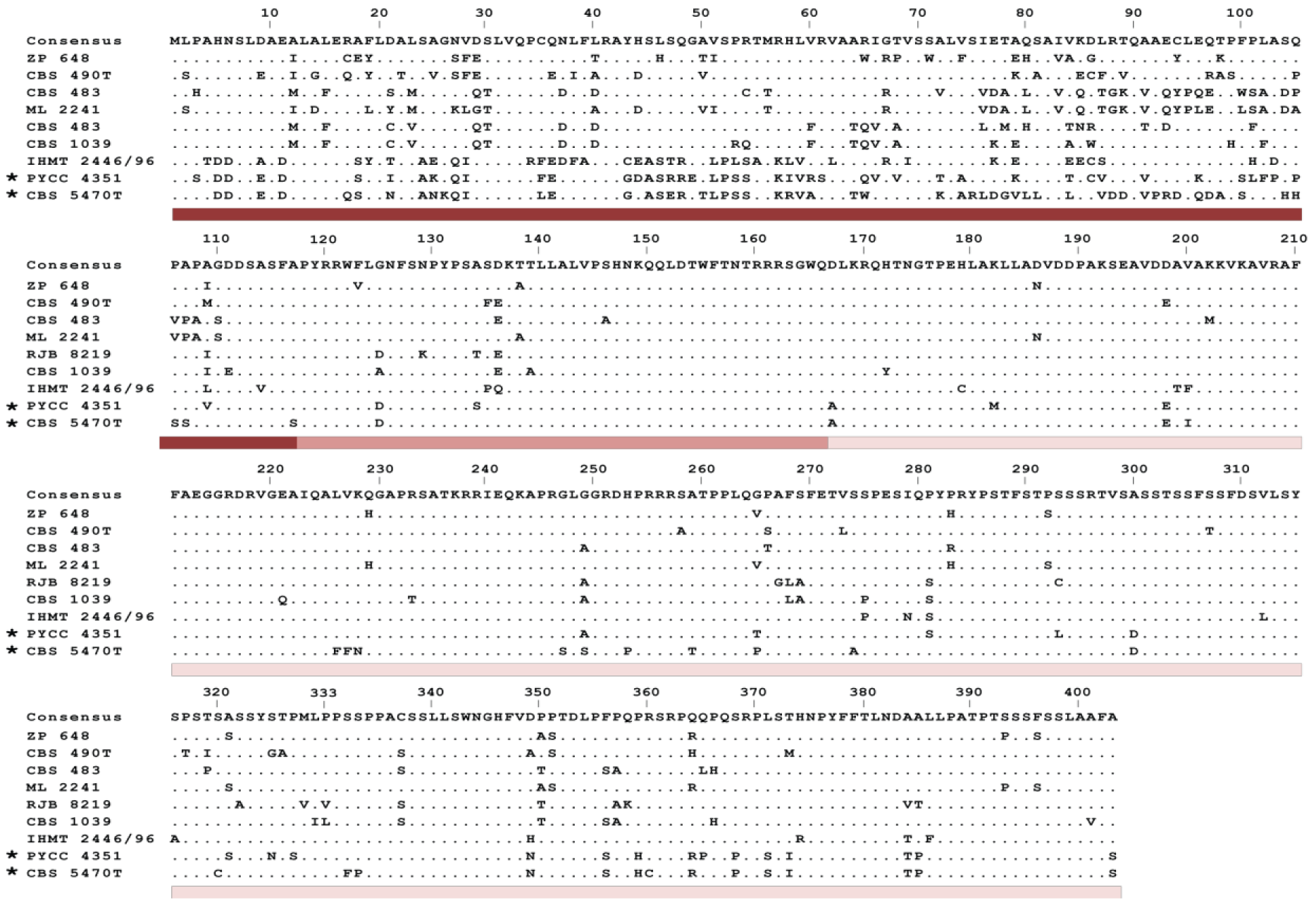

Supplement: Figure S4 — Alignment of the HD1 proteins of S. salmonicolor and S. johnsonii. Alignment of the nine deduced protein sequences used in the evolutionary rates analysis (dN/dS). Coloured bars indicate the same three domains of the HD1 protein as in Figure 2. Sequences marked with an asterisk belong to the sibling species S. johnsonii. (0.86 MB TIF) [file pgen.1001052.s004.tif]

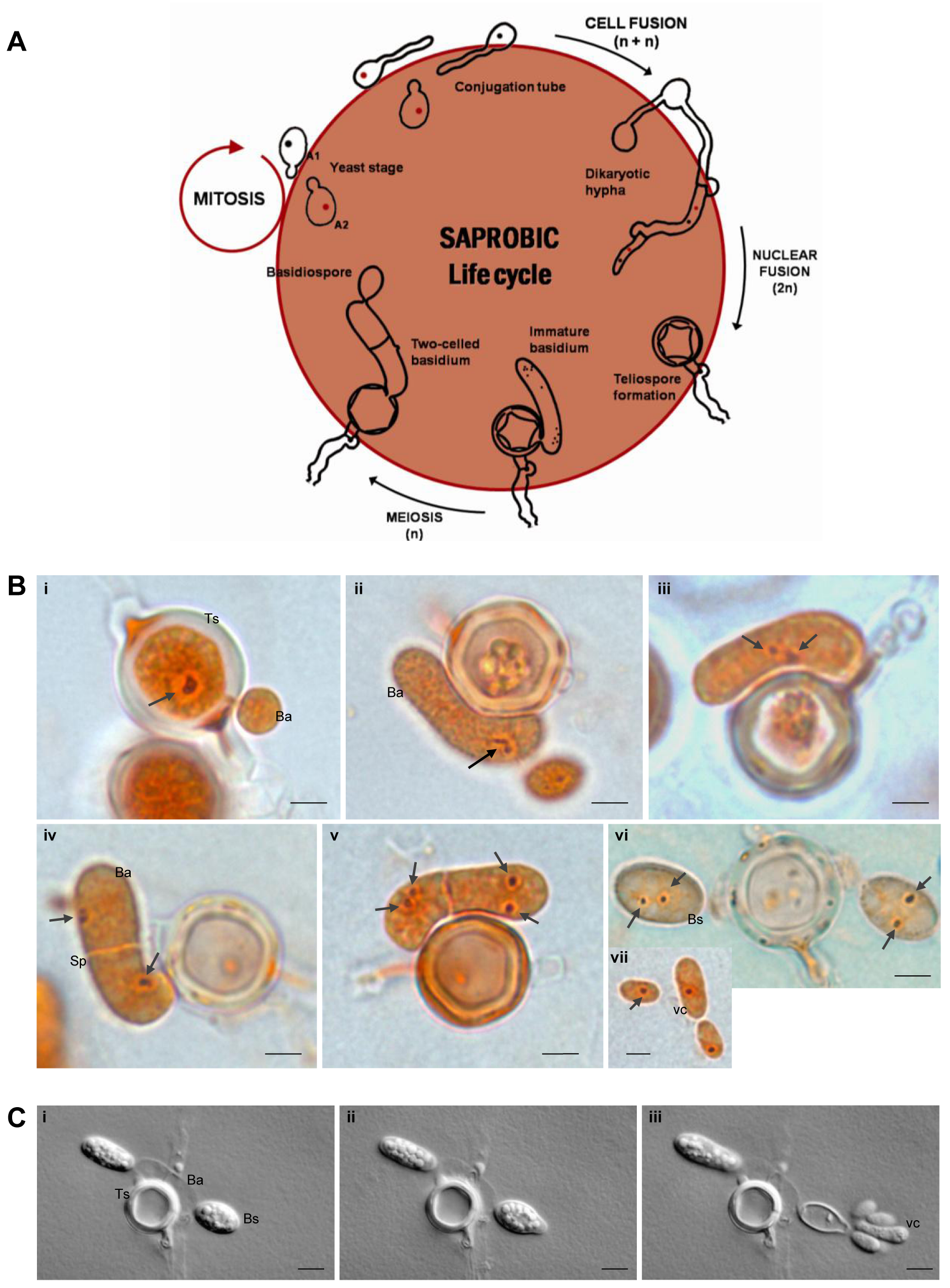

Supplement: Figure S5 — Life cycle of Sporidiobolus salmonicolor. (A) Diagrammatic representation of the saprobic life cycle of S. salmonicolor. (B) Different stages of teliospore germination and formation of basidia and basidiospores; nuclei were stained with safranin O and are indicated by arrows: (i) initial stage of teliospore [Ts] germination with diploid nucleus and basidium initial [Ba]; (ii) migration of the diploid nucleus to the immature basidium; (iii) first meiotic division; (iv) formation of septum [Sp] originating a two-celled basidium; (v) second meiotic division yielding four nuclei; (vi) formation of two large binucleated basidiospores [Bs]; (vii) haploid uninucleated vegetative yeast cells [vc] resulting from basidiospore germination. Bars = 2.5 µm. (C) Time course observation of asynchronous germination of the basidiospores (i–iii). Note that, contrary to the basidiospore on the left, the basidiospore on the right formed yeast cells. Bars = 5 µm. (5.94 MB TIF) [file pgen.1001052.s005.tif]

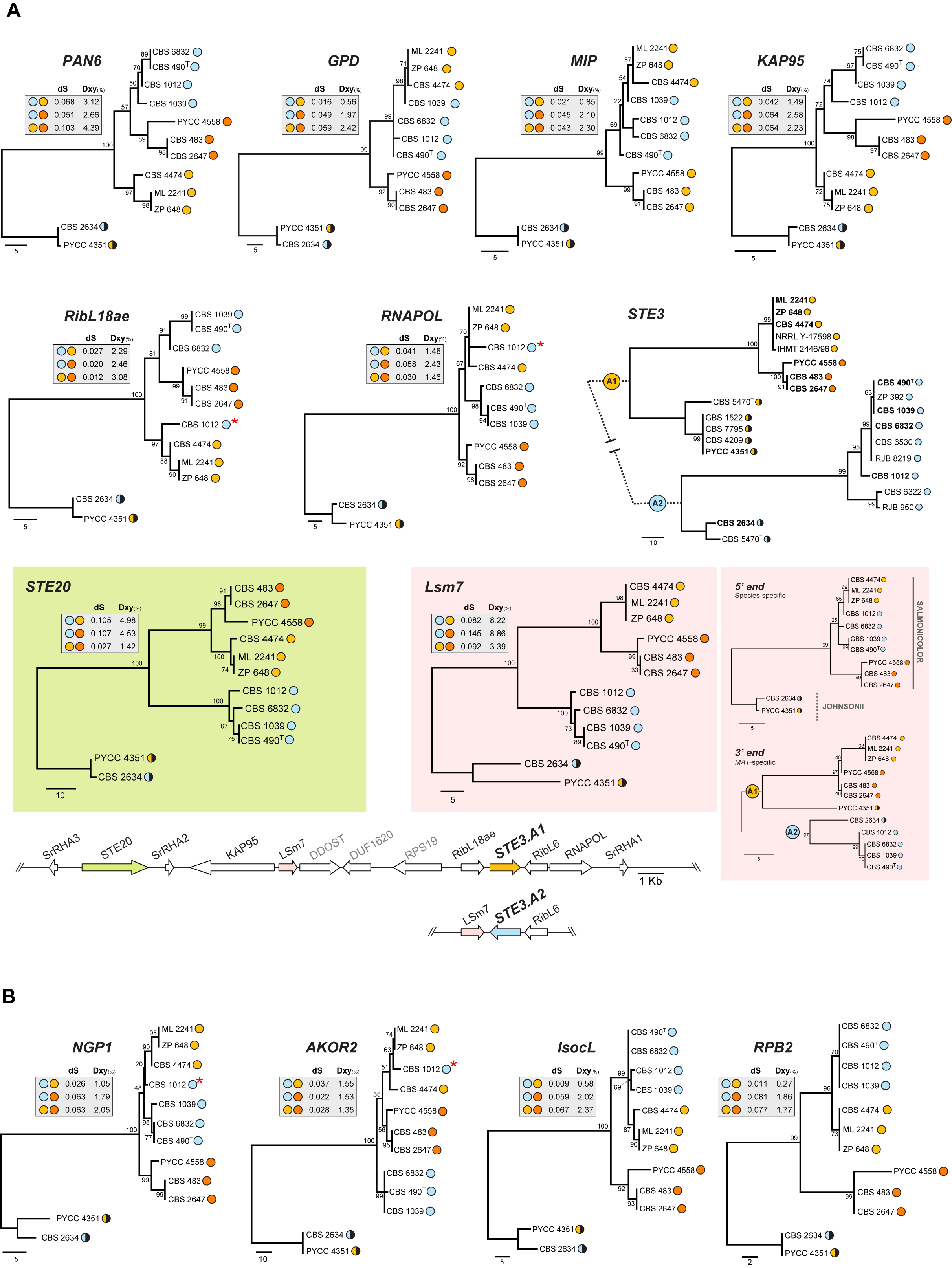

Supplement: Figure S6 — Phylogeny of several genes located in the two scaffolds harbouring MAT genes. The phylograms represent the single most likely tree for the selected genes located in the STE3 region (A) and in the HD1/HD2 region (B). Trees were generated under the close-neighbor interchange algorithm in a heuristic search. Numbers on branches indicate statistical support calculated from 1000 bootstrap replicates. Possible gene conversion events are marked with an asterisk after the strain number. For the majority of the genes, sequences of strains of MAT A2 (indicated by blue circles) are highly similar. In contrast, MAT A1 strains exhibit more sequence divergence, suggesting the existence of two lineages within this mating type (strains indicated by yellow and orange circles). Half-colored circles indicate S. johnsonii strains. Colored-shaded trees depict genes with sequence divergence clearly associated with mating type in S. salmonicolor. The position of the LSm7 gene (light pink) relative to the STE3 gene is shown for both mating types. Values for synonymous substitutions (dS) and divergence percentage (Dxy, %) are given for all genes (except for STE3) and in each pair comparisons. (1.39 MB TIF) [file pgen.1001052.s006.tif]

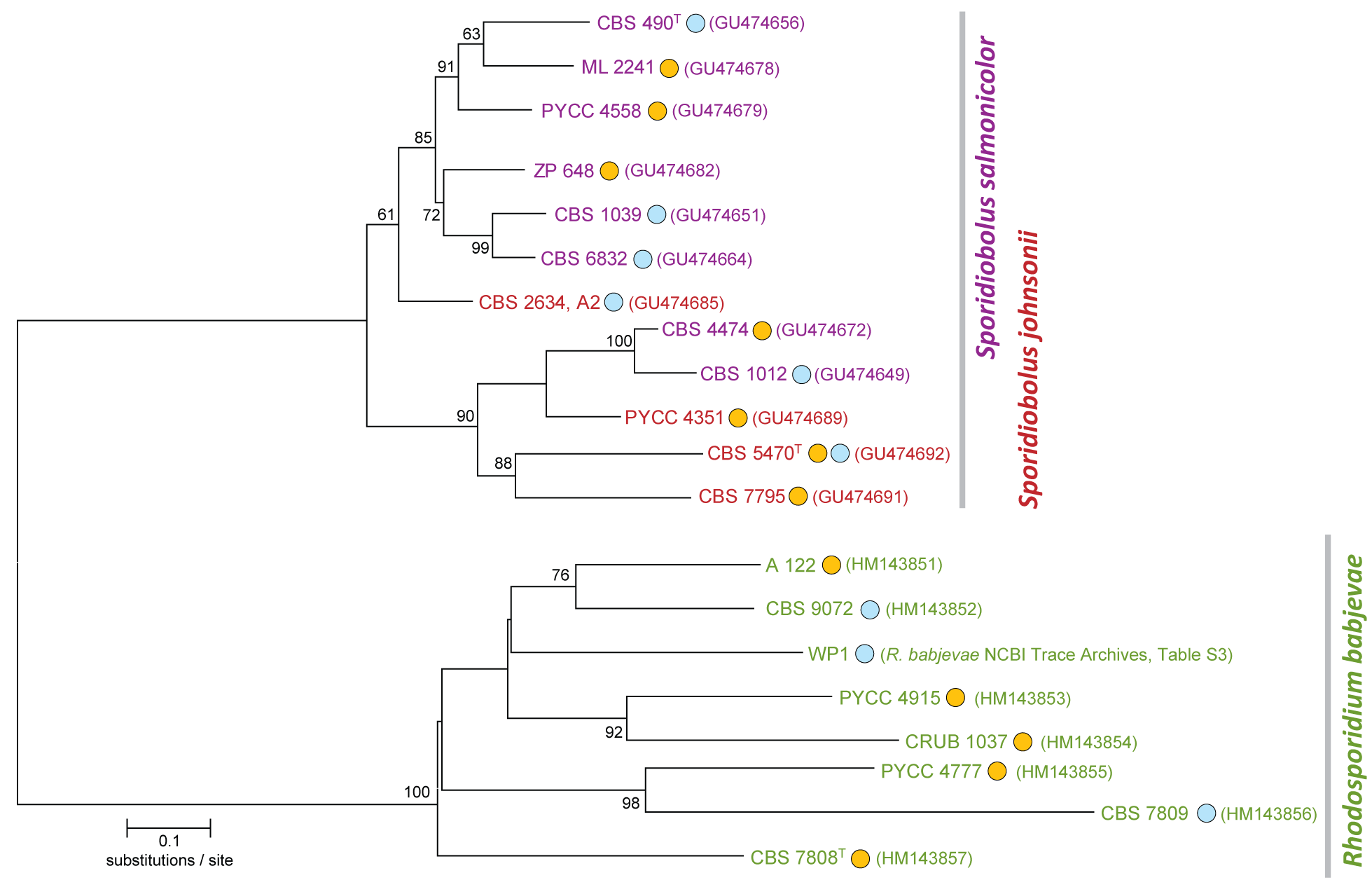

Supplement: Figure S7 — Phylogeny of HD1/HD2 alleles in Rhodosporidium babjevae. The pheromone receptor gene (STE3.A1, yellow circles; STE3.A2, blue circles) and sequence accession numbers (in brackets) are indicated after strain number. The tree was reconstructed with Neighbour-joining and TrN+G model (shape parameter = 1.68453). Bootstrap values (>50%) from 1000 replicates are shown. S. salmonicolor and S. johnsonii are included for comparison. (0.37 MB TIF) [file pgen.1001052.s007.tif]
