# Supplementary material for: A Deviation from the Bipolar-Tetrapolar Mating Paradigm in an Early Diverged Basidiomycete
Source: PLoS Genet. 2010 Aug 5;6(8):e1001052. doi: 10.1371/journal.pgen.1001052 (PMC2916851; doi:10.1371/journal.pgen.1001052)
Supplement: Table S1 — List of S. salmonicolor and S. johnsonii strains used in this study and relevant information pertaining to them. (0.73 MB PDF) [file pgen.1001052.s008.pdf]

Table S1. List of *S. salmonicolor* and *S. johnsonii* strains used in this study and relevant information pertaining to them.

Species	Strain	Sexuality[a] (mating tests)	*STE3* alleles		*HD1/HD2* alleles		Source
				GenBank accession numbers		GenBank accession numbers	
Sporidiobolus salmonicolor	CBS 1012	A2	*STE3.A2*	HM133782	A2-16	GU474649	Leaf of *Aristolochia* sp., hot-house of Botanic Laboratory, Delft, Netherlands
	CBS 1013	A2	*STE3.A2*		A2-14	GU474650	Air in dairy, USA
	CBS 1039	A2	*STE3.A2*	HM133779	A2-14	GU474651	Air in dairy, USA
	CBS 2635	A2	*STE3.A2*		A2-13	GU474652	Infected skin of a man, Germany
	CBS 2873	A2	*STE3.A2*		A2-15	GU474653	Extract of oak bark, France
	CBS 4030	A2	*STE3.A2*		A2-10	GU474654	Soil, New Zealand
	CBS 487	A2	*STE3.A2*		A2-10	GU474655	Air, Japan (type strain of *Pseudomonilia rubiduncula* Okunuki)
	CBS 490[T]	A2	*STE3.A2*	GU474641	A2-10	GU474656	Culture contaminant (type strain of *Sporidiobolus salmonicolor* Fell & Statzell Tallman)
	CBS 495	A2	*STE3.A2*		A2-13	GU474657	Culture contaminant, Japan
	CBS 496	A2	*STE3.A2*		A2-10	GU474658	Scorched skin of orange, Delft, Netherlands
	CBS 5937	A2	*STE3.A2*		A2-14	GU474659	Carious dentine, South Africa (type strain of *Aessosporon salmonicolor* van der Walt)
	CBS 6322	A2	*STE3.A2*	HM133783	A2-12	GU474660	Rotting wood shavings, Chile
	CBS 6470	A2	*STE3.A2*		A2-15	GU474661	Air, Germany
	CBS 6530	A2	*STE3.A2*	HM133780	A2-15	GU474662	Bog, locality unknown
	CBS 6781	A2	*STE3.A2*		A2-15	GU474663	Bituminous soil, oil field near Cumanà, Venezuela (type strain of *Sporidiobolus veronae* Balloni et. al.)
	CBS 6832	A2	*STE3.A2*	GU474646	A2-15	GU474664	Cerebro-spinal fluid, India (type of *Sporobolomyces salmonicolor* Kluyver & van Niel var. fischeri Misra & Randhawa)
	CBS 7260	A2	*STE3.A2*		A2-13	GU474665	Slime flux of *Quercus pendunculata*, Russia (type strain of *Sporobolomyces philippovii* Krasil'nikov)
	PYCC 5245	A2	*STE3.A2*		A2-13	GU474666	Unknown substrate, Portugal
	RJB 8219	A2	*STE3.A2*	HM133781	A2-15	GU474667	Isolated by R. J. Bandoni
	RJB 950	A2	*STE3.A2*	HM133784	A2-11	GU474668	Isolated by R. J. Bandoni (deposited at CBS as *Sporobolomyces odorus* Derx)
	ZP 392	A2	*STE3.A2*	HM133778	A2-10	GU474669	Fruit body of *Dacrymyces* sp., Germany
	CBS 2647	A1	*STE3.A1*	HM133773	A1-2	GU474670	Gut of *Drosophila* sp., locality unknown

Table S1. Continued.

Species	Strain	Sexuality[a] (mating tests)	STE3 alleles		HD1/HD2 alleles		Source
				GenBank accession numbers		GenBank accession numbers	
Sporidiobolus salmonicolor	CBS 2648	A1	*STE3.A1*		A1-5	GU474671	Exudate of *Kalopanax vicinifolium* var. *typicum*, isolated by K. Tubaki, Japan
	CBS 4474	A1	*STE3.A1*	HM133770	A1-5	GU474672	Blistered skin of a man, Bonn, Germany
	CBS 483	A1	*STE3.A1*	GU474642	A1-2	GU474673	Rusted leaf of *Citrus* sp., France (type strain of *Sporobolomyces odorus* Derx)
	CBS 497	A1	*STE3.A1*		A1-4	GU474674	Unknown (deposited at CBS as *Rhodomyces kochii*)
	CBS 6529	A1	*STE3.A1*		A1-3	GU474675	Culture contaminant, R.J. Bandoni
	IHMT 2446/96	A1	*STE3.A1*	HM133771	A1-6	GU474676	Skin lesion, Portugal
	NRRL Y-17498	A1	*STE3.A1*	HM133772	A1-3	GU474677	Ice core, Greenland
	ML 2241	A1	*STE3.A1*	GU474643	A1-3	GU474678	Isolated by J. W. Fell
	PYCC 4558	A1	*STE3.A1*	HM133774	A1-1	GU474679	Polluted river water, Oeiras, Portugal
	PYCC 4623	A1	*STE3.A1*		A1-3	GU474680	Soil, USSR
	RJB 948	A1	*STE3.A1*		A1-3	GU474681	Isolated by R. J. Bandoni (deposited at CBS as *Sporobolomyces odorus* Derx)
	ZP 648	A1	*STE3.A1*	HM133769	A1-4	GU474682	Leaf of *Ligustrum* sp., Monte de Caparica, Portugal
	CBS 2641	AS	*STE3.A2*		A2-10	GU474683	Air, Netherlands
	CBS 4029	AS	*STE3.A2*		A2-15	GU474684	Soil, New Zealand
Sporidiobolus johnsonii	CBS 2634	A2	*STE3.A2*	GU474647	A2-17	GU474685	*Fragaria sp.*, Japan (deposited at CBS as *Sporidiobolus johnsonii* Nyland)
	CBS 2630	A1	*STE3.A1*		A1-9	GU474686	Air, The Netherlands
	CBS 2643	A1	*STE3.A1*		A1-9	GU474687	Isolated by S. Windisch, Germany
	CBS 4209	A1	*STE3.A1*	HM133776	A1-8	GU474688	Fruit body of *Exidia* sp., Japan (type strain of *Sporobolomyces coralliformis* Tubaki)
	PYCC 4351	A1	*STE3.A1*	GU474644	A1-9	GU474689	Leaf of a tree, Portugal
	CBS 1522	AS	*STE3.A1*	HM133775	A1-9	GU474690	Fodder yeast, Germany (type strain of *Sporobolomyces holsaticus* Windish)
	CBS 7795	AS	*STE3.A1*	HM133777	A1-7	GU474691	Culture contaminant, isolated by W. I. Golubev, Russia
	CBS 5470[T]	SF	*STE3.A1*<br>*STE3.A2*	GU474645<br>GU474648	A1/A2-18	GU474692	Leaf of *Rubus idaeus* with dead pustule of *Phragmidium rubi-idaei*, USA (type strain of *Sporidiobolus johnsonii* Nyland)
	CBS 8241	SF	*STE3.A1*<br>*STE3.A2*		A1/A2-18	GU474693	Unknown

[a]The mating type designations (A1 or A2) were re-assigned to match the "molecular mating type" identified by PCR detection of the pheromone receptor alleles (*STE3.A1 or STE3.A2*).
