# Supplementary material for: A Deviation from the Bipolar-Tetrapolar Mating Paradigm in an Early Diverged Basidiomycete
Source: PLoS Genet. 2010 Aug 5;6(8):e1001052. doi: 10.1371/journal.pgen.1001052 (PMC2916851; doi:10.1371/journal.pgen.1001052)
Supplement: Table S2 — List of primers, specific PCR conditions, and restriction enzymes used for the RFLP analysis. (0.50 MB PDF) [file pgen.1001052.s009.pdf]

Table S2. List of primers, specific PCR conditions and restriction enzymes used for the RFLP analysis.

Primer Name	Sequence (5' → 3')	Annealing/ Extension	Purpose	Restriction enzyme used in RFLP analysis
**MC122**<br>**MC123**	TGGCGMGTYCCCGAACCYTACAAC<br>AAAGGSTACGACCAGYTSCTCAAYCT	62.5ºC / 3 min	Amplify the complete ***STE3.A2*** **allele** in the type strain of *Ss* (CBS 490^T)	
**MC126**<br>**MC127**	AATRCCGCTCCGCTYTACTGGCA<br>TGTTCGGGTCTACAGGTTGGAGAG	62ºC / 90 s		
**MC103**<br>**MC104**	AGGATGTCGAGGACTTCTTCGGTGA<br>CGGCGCGTGTTGGTGAACCAGGT	62 ºC / 90 s	Amplify the **HD1/HD2 region** in all the available strains of *Ss* and *Sj*	Rsa I
**MC053**<br>**MC054**	CATCTTCTTCCTCCTCCTGCC<br>GGGTAGGGAGGAAGCGAGAT	60ºC / 90 s	Screening the ***STE3.A1*** and ***STE3.A2*** alleles in all the available strains of *Ss* and *Sj*	
**MC126**<br>**MC127**	AATRCCGCTCCGCTYTACTGGCA<br>TGTTCGGGTCTACAGGTTGGAGAG	62ºC / 90 s		
**MC111**<br>**MC112**	CGCCGCTGGTTCCTTGRCAACTTCT<br>GCTGCCCATCTACGAGCAAGTCCC	62ºC / 90 s	Amplify the C-terminal region of the ***HD1*** gene in selected *Ss* and *Sj* strains	
**MC147**<br>**MC148**	GARGACCTCGCSACCTACCC<br>AGGAKGAGMGCTTGYTGGATGT	60ºC / 30 s	Amplify part of the ***PAN6*** gene in *Ss*	Alu I
**MC045**<br>**MC046**	CGMGAAGACCTGAARCCGTT<br>CATGWACTCCATSACGACCCA	56ºC / 90 s	Amplify part of the ***STE20*** gene in *Ss*	Eco RI
**MC131**<br>**MC132**	CGAYATCRCCRAAACAGCTGAGGAT<br>TACAACGARCTCCARGTCAACATCT	59ºC / 90 s	Amplify part of the ***KAP95*** gene in *Ss*	
**MC133**<br>**MC134**	ACCTCAGCAAGTACGTYGACAAGAA<br>AAAGGGTTTGCAATTTCCKCMGAC	59ºC / 90 s	Amplify part of the ***LSm7*** gene in *Ss*	Rsa I
**MC135**<br>**MC136**	GAGAAGGAGCCKCARCCCAA<br>ACATCAAGCAGCTCCTCACCC	56ºC / 90 s	Amplify part of the ***RibL18*** gene in *Ss*	Kpn I
**MC137**<br>**MC138**	GACTTCCCRGGYCACTAYCCC<br>CCTGTCCKCCKCGCATCTT	59ºC / 90 s	Amplify part of the ***RNAPOL*** gene in *Ss*	
**MC141**<br>**MC142**	CTACATGTTCAAGTACGACTCGAC<br>GTACCACGAGAAGAGCTTGACGAA	59ºC / 90 s	Amplify part of the ***GPD*** gene in *Ss*	Stu I
**MC139**<br>**MC140**	TCGATCCTCATGGAGCAYTTCATCT<br>CCAYTTGCCMACYTCTTCCATC	59ºC / 90 s	Amplify part of the ***MIP*** gene in *Ss*	
**MC157**<br>**MC158**	GTCTTGACGACCGGGTTCAACGA<br>GGCAWTCCKCCTCGGTAGTGGTA	59ºC / 90 s	Amplify part of the ***IsocL*** gene in *Ss*	Bam HI
**MC155**<br>**MC156**	TCAACMTCCTCTCYGGCAAGTC<br>GAGTTRTTGATGGAGGCGTGGAA	59ºC / 90 s	Amplify part of the ***NGP1*** **(*NOG2*)** gene in *Ss*	
**MC149**<br>**MC150**	CTGGACARGAGACGTACAATGC<br>TCTCGAGCRTGAARTCAAAGACGTT	59ºC / 90 s	Amplify part of the ***AKOR2*** gene in *Ss*	Rsa I
**MC151**<br>**MC152**	CTGAACCGMTACACCTTTGCC<br>TGAATCTCGCAATGWGTCCAAG	59ºC / 1 min	Amplify part of the ***RPB2*** gene in *Ss*	

S. salmonicolor **(Ss)**; ***S. johnsonii*** **(*Sj*)**

Table S2. Continued.

Primer Name	Sequence (5' → 3')	Annealing/ Extension	Purpose	Restriction enzyme used in RFLP analysis
**MC170**<br>**MC171**	TATCTACGGCGCTGGTTGCTTGA<br>TCCAGATCATCGAGCGGTCCTTG	60 ºC / 90 s	Amplify part of the ***sdhA*** gene in *Ss*	
**MC047**<br>**MC048**	ACCAACCTYTGCGTYTCRGTCGA<br>GATGACCTCGTCSGGYGTACKGTA	63 ºC / 90 s	Amplify part of the ***URA3*** gene in *Ss*	
**MC172**<br>**MC173**	GCCAGTCCCTCATCACCTCG<br>TCCTTGAGCCTGAAGCAGTCG	60ºC / 90 s	Amplify part of the ***HXT1*** gene in *Ss*	
**MC174**<br>**MC175**	GACCAGCAGTTCTTGGCCGA<br>GAGCTTGACAAACTCGTCGTGAAC	60ºC / 90 s	Amplify part of the ***aldA*** gene in *Ss*	
**MC176**<br>**MC177**	AGCGTTGCGTTCCTCCAGTC<br>GTTCTTGGCGACCTCGATCTG	60ºC / 90 s	Amplify part of the ***PAL*** gene in *Ss*	
**MC178**<br>**MC179**	CTCATCATCGTCGACTCTGTCAC<br>GGCGAGTCGACGATCTTGG	60ºC / 60 s	Amplify part of the ***DMC1*** gene in *Ss*	Msp I
**MC180**<br>**MC181**	GCCGTCTACCGAATCAACAAG<br>CTGCATCTGGAGCTTGCATTT	57ºC / 90 s	Amplify part of the ***GEF1*** gene in *Ss*	
**MC182**<br>**MC183**	CCCATCCTGAATGTCAACTACAA<br>GGTGACAAGAACCGAGTAGCG	58ºC / 90 s	Amplify part of the ***LACC*** gene in *Ss*	
**MC186**<br>**MC187**	CAAGTGCCTGTGCAAGAA<br>CATAAGCCATGTACGGTTT	51ºC / 60 s	Amplify part of the ***RAN1*** gene in *Ss*	Hinf I

S. salmonicolor **(Ss);** ***S. johnsonii*** **(*Sj*)**
