# Supplementary material for: A Deviation from the Bipolar-Tetrapolar Mating Paradigm in an Early Diverged Basidiomycete
Source: PLoS Genet. 2010 Aug 5;6(8):e1001052. doi: 10.1371/journal.pgen.1001052 (PMC2916851; doi:10.1371/journal.pgen.1001052)
Supplement: Table S3 — NCBI Trace Archives sequences used in this study. (0.73 MB PDF) [file pgen.1001052.s010.pdf]

Table S3. NCBI Trace Archives sequences used in this study.

Rhodosporidium babjevae	
STE3.A2 (includes the 5’-end of LSm7 and 3’-end of RibL6 putative flanking genes)	
ti| **2154151284**	name:FUHB15613.g1
ti| **2195334063**	name:FWAF22906.b1
ti| **2195327270**	name:FWAF17973.g1
ti| **2154446490**	name:FWAG21685.b1
ti| **2154537287**	name:FWAG43948.b1
ti| **2154452092**	name:FWAG24486.b1
ti| **2154291912**	name:FUHB42794.g1
ti| **2154443469**	name:FWAG20174.g1
Sporidiobolus salmonicolor	
HD1/HD2 region	
ti| **1244034137**	name:BAUG63963.g1
ti| **1243919455**	name:BAUG5057.g1
ti| **1244020867**	name:BAUG56453.b1
ti| **1243931890**	name:BAUG10196.g1
ti| **1243965174**	name:BAUG37624.g1
ti| **1244003711**	name:BAUG55425.b1
ti| **1243998420**	name:BAUG42358.g1
ti| **1244025540**	name:BAUG59590.g1
ti| **1244020332**	name:BAUG49966.b1
ti| **1243943891**	name:BAUG16629.b1
ti| **1243937469**	name:BAUG10687.g1
ti| **1243958334**	name:BAUG25600.b1
ti| **1244003699**	name:BAUG55413.b1
ti| **1243967269**	name:BAUG34439.g1
ti| **1243996862**	name:BAUG45312.b1
ti| **1244031458**	name:BAUG63684.g1
ti| **1243974274**	name:BAUG32324.g1
ti| **1243951950**	name:BAUG19696.b1
ti| **1243993653**	name:BAUG42295.b1
**Partial sequence of the *RPO41* gene** (Putative DNA-dependent RNA polymerase)	
ti| **1243937005**	name:BAUG11471.b1
ti| **1243946449**	name:BAUG18899.b1
ti| **1243925713**	name:BAUG9587.g1
ti| **1244026558**	name:BAUG59648.g1
ti| **1243980948**	name:BAUG37078.b1
ti| **1244022107**	name:BAUG60765.b1
ti| **1243999058**	name:BAUG38580.g1
ti| **1244030807**	name:BAUG63417.b1
**Partial sequence of the *PAN6* gene** (Putative pantoate-beta-alanine ligase)	
ti| **1243999491**	name:BAUG39589.b1
ti| **1243944265**	name:BAUG18155.g1
ti| **1243939898**	name:BAUG15996.b1
ti| **1244024936**	name:BAUG62826.g1
ti| **1243921720**	name:BAUG5210.b1

**Partial sequence of the *STE20* gene** (Putative p21-activated protein kinase)	
ti| **1243947537**	name:BAUG24979.g1
ti| **1243983780**	name:BAUG45862.b1
ti| **1244033153**	name:BAUG61059.b1
ti| **1243931275**	name:BAUG14477.g1
ti| **1243964000**	name:BAUG19266.b1
ti| **1244010134**	name:BAUG51096.b1
ti| **1244008391**	name:BAUG52617.b1
ti| **1244012976**	name:BAUG51538.g1
ti| **1243932804**	name:BAUG11782.g1
ti| **1244008568**	name:BAUG52794.b1
ti| **1243986237**	name:BAUG46207.g1
ti| **1243931275**	name:BAUG14477.g1
**Partial sequence of the *KAP95* gene** (Putative Karyopherin β 1 - importin)	
ti| **1243914633**	name:BAUG2422.x1
ti| **1243945047**	name:BAUG18361.g1
ti| **1244013243**	name:BAUG52477.b1
ti| **1244003594**	name:BAUG55308.b1
ti| **1243947825**	name:BAUG24979.b1
ti| **1244019354**	name:BAUG53116.g1
ti| **1244010118**	name:BAUG51080.b1
ti| **1244023708**	name:BAUG62750.b1
ti| **1243943827**	name:BAUG18773.b1
ti| **1244005440**	name:BAUG49762.b1
**Partial sequence of the *LSm7* gene** (Putative small nuclear ribonucleoprotein)	
ti| **1243966515**	name:BAUG34933.b1
ti| **1244006137**	name:BAUG55067.b1
ti| **1244019455**	name:BAUG53217.g1
ti| **1244006358**	name:BAUG55288.b1
ti| **1243968129**	name:BAUG36451.g1
ti| **1244035821**	name:BAUG58639.g1
**Partial sequence of the *RibL18* gene** (Putative Ribosomal L18ae protein)	
ti| **1244029876**	name:BAUG59030.g1
ti| **1244027010**	name:BAUG60100.b1
ti| **1243938823**	name:BAUG14825.b4
ti| **1244024596**	name:BAUG61718.b1
**Partial sequence of the *RNAPOL* gene** (Putative DNA directed RNA polymerase III, 30/40 KDa subunit)	
ti| **1243928029**	name:BAUG8159.b1
ti| **1244017874**	name:BAUG56436.b5
ti| **1243938919**	name:BAUG14825.g2
ti| **1244027106**	name:BAUG60100.g1
ti| **1244002511**	name:BAUG47217.g1
**Partial sequence of the *GPD* gene** (Putative glyceraldehyde-3-phosphate dehydrogenase – GAPDH)	
ti| **1244012762**	name:BAUG53916.b1
ti| **1243987204**	name:BAUG43814.b1
ti| **1243961274**	name:BAUG24220.b1

Table S3. Continued.

**Partial sequence of the *MIP* gene** (Putative mitochondrial intermediate peptidase)		
ti|	**1243979024**	name:BAUG29298.g1
ti|	**1243946492**	name:BAUG18942.b1
ti|	**1243993537**	name:BAUG42179.g1
ti|	**1244034662**	name:BAUG64392.b1
ti|	**1244008528**	name:BAUG52754.b1
ti|	**1243938225**	name:BAUG11059.g1
ti|	**1243918485**	name:BAUG1303.y1
ti|	**1243959176**	name:BAUG22218.b1
ti|	**1243964071**	name:BAUG19337.b1
**Partial sequence of *IsocL* gene** (Putative isocitrate lyase)		
ti|	**1244032592**	name:BAUG59346.g1
ti|	**1243913568**	name:BAUG1741.x1
ti|	**1243931944**	name:BAUG10250.g1
ti|	**1243952262**	name:BAUG19816.g1
ti|	**1243963834**	name:BAUG20636.b1
ti|	**1243965732**	name:BAUG38182.g2
ti|	**1243981027**	name:BAUG37061.g1
ti|	**1243971150**	name:BAUG29488.b2
**Partial sequence of the *NGP1 (NOG2)* gene** (Putative Nucleolar GTP-binding protein 2)		
ti|	**1243932700**	name:BAUG11678.g1
ti|	**1244009264**	name:BAUG57426.b1
ti|	**1244000123**	name:BAUG39933.b1
ti|	**1243941162**	name:BAUG17164.b1
ti|	**1244019186**	name:BAUG52180.g2
ti|	**1243984108**	name:BAUG45710.g1
ti|	**1243948626**	name:BAUG24724.b1
ti|	**1244029720**	name:BAUG58970.b1
ti|	**1243918656**	name:BAUG1474.y1
ti|	**1244014951**	name:BAUG53321.b1
ti|	**1243966796**	name:BAUG33966.b1
ti|	**1243966495**	name:BAUG34913.b1
ti|	**1243933163**	name:BAUG12429.b1
ti|	**1244034658**	name:BAUG64388.b1
ti|	**1243921840**	name:BAUG5330.b1
**Partial sequence of the *AKOR2* gene** (Putative aldo-keto reductase)		
ti|	**1244016242**	name:BAUG56724.g1
ti|	**1243951077**	name:BAUG20935.b1
ti|	**1243949884**	name:BAUG28574.b1
ti|	**1244024160**	name:BAUG57922.g1
ti|	**1244021707**	name:BAUG48845.b1
ti|	**1244024160**	name:BAUG57922.g1
ti|	**1243966759**	name:BAUG33929.b1
ti|	**1243913061**	name:BAUG1233.x1
ti|	**1243949884**	name:BAUG28574.b1

**Partial sequence of the *RPB2* gene** (Putative RNA polymerase II second largest subunit)		
ti|	**1243971757**	name:BAUG32111.g1
ti|	**1244020882**	name:BAUG56468.b1
ti|	**1243983637**	name:BAUG45719.b1
ti|	**1243920596**	name:BAUG3510.g1
ti|	**1243971149**	name:BAUG29487.b2
ti|	**1243965032**	name:BAUG37578.b1
ti|	**1243961708**	name:BAUG22830.g1
ti|	**1244035646**	name:BAUG58464.g1
ti|	**1243917934**	name:BAUG6120.x1
ti|	**1243920588**	name:BAUG3502.g1
ti|	**1243922988**	name:BAUG8878.b1
ti|	**1243938766**	name:BAUG14768.g2
ti|	**1243961708**	name:BAUG22830.g1
**"Autosomal" markers – *S. salmonicolor***		
**Partial sequence of the *sdhA* gene** (Putative succinate dehydrogenase flavoprotein subunit)		
ti|	**1243932965**	name:BAUG12327.b1
ti|	**1243949976**	name:BAUG28570.g1
ti|	**1244011373**	name:BAUG57519.g1
ti|	**1243982038**	name:BAUG29912.b1
ti|	**1244024325**	name:BAUG60967.g1
**Partial sequence of the *URA3* gene** (orotidine 5'-phosphate decarboxylase)		
ti|	**1244022690**	name:BAUG61732.g1
ti|	**1244004916**	name:BAUG55766.g1
ti|	**1243996898**	name:BAUG44964.g1
ti|	**1243942104**	name:BAUG17626.b1
ti|	**1243939980**	name:BAUG15982.g1
ti|	**1243942104**	name:BAUG17626.b1
ti|	**1244011207**	name:BAUG57353.g1
**Partial sequence of the *HXT1* gene** (putative hexose transporter)		
ti|	**1243923129**	name:BAUG8923.g1
ti|	**1243923641**	name:BAUG9147.g1
ti|	**1244007487**	name:BAUG48257.b1
ti|	**1243978746**	name:BAUG37660.g1
ti|	**1244008309**	name:BAUG49847.g1
ti|	**1243932413**	name:BAUG11391.g1
ti|	**1244025679**	name:BAUG59729.b1
ti|	**1244028271**	name:BAUG62513.b2
ti|	**1243928371**	name:BAUG9653.b1
ti|	**1243949421**	name:BAUG24847.b1
ti|	**1243981984**	name:BAUG29858.b1
ti|	**1244014361**	name:BAUG54075.g1
ti|	**1243923622**	name:BAUG9128.g1
ti|	**1243953511**	name:BAUG23465.g1
ti|	**1243935956**	name:BAUG14166.g1
ti|	**1243943748**	name:BAUG18694.b1
ti|	**1244021128**	name:BAUG56714.b1
ti|	**1243914662**	name:BAUG2451.x1
ti|	**1243923263**	name:BAUG8961.g1
ti|	**1244019696**	name:BAUG53650.b1
ti|	**1243916660**	name:BAUG4840.y1
ti|	**1243927197**	name:BAUG7711.g1

Table S3. Continued.

**Partial sequence of the *RAN1* gene** (putative protein serine/ threonine kinase)		
ti|	**1243988647**	name:BAUG46505.b1
ti|	**1244016377**	name:BAUG48987.b1
ti|	**1244006386**	name:BAUG55796.g1
ti|	**1244005972**	name:BAUG52982.g1
ti|	**1243915389**	name:BAUG2796.x1
ti|	**1243988647**	name:BAUG46505.b1
**Partial sequence of the *aldA* gene** (putative NAD-aldehyde dehydrogenase)		
ti|	**1244020809**	name:BAUG56203.b2
ti|	**1244016587**	name:BAUG51021.g1
ti|	**1243935797**	name:BAUG14103.g1
ti|	**1243978923**	name:BAUG29197.g1
ti|	**1243958883**	name:BAUG21445.b1
ti|	**1243988822**	name:BAUG46584.b1
ti|	**1243930459**	name:BAUG12893.g1
ti|	**1244032672**	name:BAUG59426.b1
ti|	**1244033401**	name:BAUG61211.b1
ti|	**1244014959**	name:BAUG53329.b1
ti|	**1243978923**	name:BAUG29197.g1
ti|	**1243986112**	name:BAUG46178.b1
ti|	**1244010416**	name:BAUG51282.b1
ti|	**1243964402**	name:BAUG20436.g1
ti|	**1244032672**	name:BAUG59426.b1
ti|	**1244033401**	name:BAUG61211.b1
ti|	**1244014959**	name:BAUG53329.b1
ti|	**1243978923**	name:BAUG29197.g1
ti|	**1243986112**	name:BAUG46178.b1
ti|	**1244010416**	name:BAUG51282.b1
ti|	**1243964402**	name:BAUG20436.g1
**Partial sequence of the *PAL* gene** (putative NAD-aldehyde dehydrogenase)		
ti|	**1244023897**	name:BAUG62939.b1
ti|	**1243917414**	name:BAUG5594.y1
ti|	**1244028263**	name:BAUG62505.b2
ti|	**1243999288**	name:BAUG38714.b1
ti|	**1243988576**	name:BAUG45666.b1
ti|	**1243941753**	name:BAUG15739.g1
ti|	**1243997975**	name:BAUG39225.g1
ti|	**1243979468**	name:BAUG29742.b1
**Partial sequence of the *DMC1* gene** (putative meiosis-specific recombinase)		
ti|	**1244026568**	name:BAUG59658.g1
ti|	**1243954225**	name:BAUG23891.g1
ti|	**1244015333**	name:BAUG50055.g1
ti|	**1244030098**	name:BAUG58388.b1
**Partial sequence of the *GEF1* gene** (putative phenylalanine ammonia-lyase)		
ti|	**1243992869**	name:BAUG42087.b1
ti|	**1243919507**	name:BAUG5109.g1
ti|	**1243920956**	name:BAUG6942.g1
ti|	**1243975744**	name:BAUG33506.g1
ti|	**1243981595**	name:BAUG30237.b1
ti|	**1243982772**	name:BAUG29782.g1
ti|	**1244026095**	name:BAUG60337.g1
ti|	**1243982553**	name:BAUG30715.g1

**Partial sequence of the *LACC* gene** (putative laccase protein)		
ti|	**1244008544**	name:BAUG52770.b1
ti|	**1243941934**	name:BAUG17456.b1
ti|	**1243992777**	name:BAUG41995.g1
ti|	**1243956635**	name:BAUG27165.b1
ti|	**1244018080**	name:BAUG50306.g1
ti|	**1243928557**	name:BAUG12047.b1
ti|	**1244013399**	name:BAUG53785.g1
ti|	**1243954711**	name:BAUG24185.b1

Rhodosporidium babjevae		
HD1-HD2		
ti|	**2154331804**	name:FWAF8985.g1
ti|	**2154456123**	name:FWAG26501.g1
ti|	**2154540940**	name:FWAG45774.g1
ti|	**2154229020**	name:FUHB29492.b1
ti|	**2195319826**	name:FWAF13483.g1
ti|	**2154235755**	name:FUHB32859.g1
ti|	**2195334783**	name:FWAF24034.b1
ti|	**2154503699**	name:FWAG36226.b1
ti|	**2195316925**	name:FWAF12033.b1
ti|	**2154229021**	name:FUHB29492.g1
ti|	**2154430327**	name:FWAG18979.b1
ti|	**2195318461**	name:FWAF12801.b1
ti|	**2154533013**	name:FWAG41811.b1
ti|	**2154534672**	name:FWAG42640.g1
ti|	**2154333225**	name:FWAF994.b1
ti|	**2195322807**	name:FWAF15742.b1
ti|	**2195332082**	name:FWAF20763.g1
ti|	**2154197503**	name:FUHB28724.g1
ti|	**2154499347**	name:FWAG34050.b1
ti|	**2195335759**	name:FWAF24522.b1
ti|	**2154197502**	name:FUHB28724.b1
ti|	**2154447297**	name:FWAG22088.g1
ti|	**2154500783**	name:FWAG34768.b1
ti|	**2154233232**	name:FUHB31598.b1
ti|	**2154419698**	name:FWAG13664.g1
ti|	**2154326269**	name:FWAF647.b1
ti|	**2154367586**	name:FWAG1711.b1
ti|	**2154430851**	name:FWAG9641.b1
ti|	**2154233233**	name:FUHB31598.g1
ti|	**2154343709**	name:FWAF10244.g1
ti|	**2154368702**	name:FWAG2269.b1
ti|	**2154321820**	name:FWAF4447.g1
ti|	**2154418949**	name:FWAG13290.b1
